# C–C (alkynylation) vs C–O (ether) bond formation under Pd/C–Cu catalysis: synthesis and pharmacological evaluation of 4-alkynylthieno[2,3-*d*]pyrimidines

**DOI:** 10.3762/bjoc.7.44

**Published:** 2011-03-21

**Authors:** Dhilli Rao Gorja, K Shiva Kumar, K Mukkanti, Manojit Pal

**Affiliations:** 1 Institute of Life Sciences, University of Hyderabad Campus, Gachibowli, Hyderabad-500 046, India; 2 Chemistry Division, Institute of Science and Technology, JNT University, Kukatpally, Hyderabad 500 072, India

**Keywords:** catalysis, C–C bond, copper, palladium, thieno[2,3-*d*]pyrimidine

## Abstract

The Pd/C–CuI–PPh_3_ catalytic system facilitated C–C bond formation between 4-chlorothieno[2,3-*d*]pyrimidines and terminal alkynes in methanol with high selectivity without generating any significant side products arising from C–O bond formation between the chloro compounds and methanol. A variety of novel 4-alkynylthieno[2,3- *d*]pyrimidines were prepared via alkynylation of 4-chlorothieno[2,3-*d*]pyrimidines in good to excellent yields. Some of the compounds synthesized were tested for cytotoxic activity in vitro.

## Introduction

**Figure 1:**
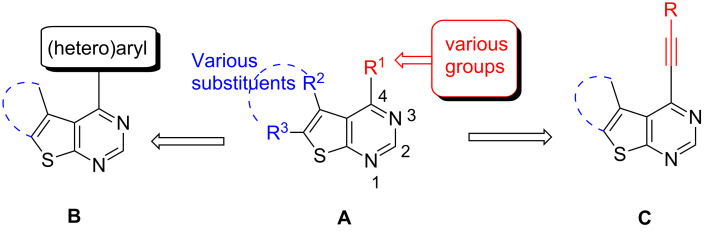
Diversity-based thieno[2,3-*d*]pyrimidine scaffold [7].

Alkynyl substituted pyrimidines are of considerable pharmacological interest because of their notable biological activities [1], in particular, adenosine kinase inhibitory activity in the treatment of pain and inflammatory diseases [2] and thymidylate synthase inhibitory properties in cancer therapy [3]. On the other hand, the thiophene moiety is a common feature in many bioactive agents and drugs [4] and is considered as a bioisostere of the benzene ring [5]. Thus, one can anticipate that combining the pyrimidine ring of an alkynyl substituted pyrimidine moiety with a thiophene ring might afford compounds, i.e., alkynyl substituted thienopyrimidines of potential pharmacological interest. Notably, 6-ethynylthieno[3,2-*d*]- and 6-ethynylthieno[2,3-*d*]pyrimidin-4-aniline derivatives were found to be potent inhibitors of ErbB family receptor tyrosine kinases (EGFR, ErbB-2) and the proliferation of tumor cells that highly express these kinases [6]. In continuation of our research program into new drug discovery, we became interested in the generation of a small-molecule library **A** (Figure 1) based on thieno[2,3-*d*]pyrimidine for in-house pharmacological evaluation. Accordingly, we recently reported the synthesis of 4-(hetero)aryl substituted thieno[2,3-*d*]pyrimidines **B** [7]. As a further extension of this research and in view of possible pharmacological value of compounds containing alkyne, thiophene and pyrimidine moieties, we now wish to report the synthesis and in vitro cytotoxicity of novel 4-alkynylthieno[2,3-*d*]pyrimidines **C** (Figure 1). These derivatives are attractive due to the synthetic potential of C-4 alkynyl fragments for further use in library construction.

**Scheme 1:**
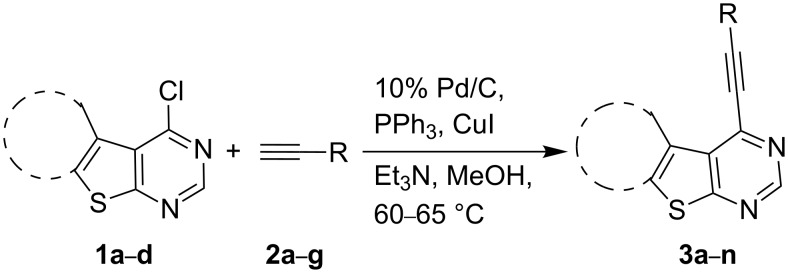
Pd/C-mediated synthesis of 4-alkynyl-substituted thieno[2,3-*d*]pyrimidines.

A number of methods have been reported for the synthesis of alkynyl substituted pyrimidines and most of which involve the use of Sonogashira coupling of halopyrimidines with terminal alkynes [1–3] (for a review see [8]). While alkynylation of the thiophene ring of thienopyrimidines under Sonogashira conditions [9] has previously been reported [6], a similar coupling reaction on the pyrimidine ring of thieno[2,3-*d*]pyrimidines is uncommon in the literature [10]. The use of Pd/C–CuI–PPh_3_ as a less expensive catalyst system for efficient Sonogashira coupling has been explored earlier. Due to our continuing interest in Pd/C-mediated alkynylation of aryl and heteroaryl halides we decided to investigate the Pd/C-based methodology for the synthesis of our target compounds, i.e., 4-alkynylthieno[2,3-*d*]pyrimidines as shown in Scheme 1.

## Results and Discussion

**Scheme 2:**
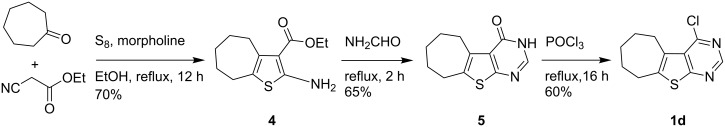
Preparation of 4-chloro-6,7,8,9-tetrahydro-5*H*-cyclohepta[4,5]thieno[2,3-*d*]pyrimidine **1d**.

The key starting materials, i.e., 4-chlorothieno[2,3-*d*]pyrimidines **1a–c** required for our synthesis were prepared according to a known method reported earlier [7]. The other chloro compound 4-chloro-6,7,8,9-tetrahydro-5*H*-cyclohepta[4,5]thieno[2,3-*d*]pyrimidine (**1d**) was prepared from cycloheptanone and ethyl cyanoacetate following a similar procedure as shown in Scheme 2.

**Scheme 3:**
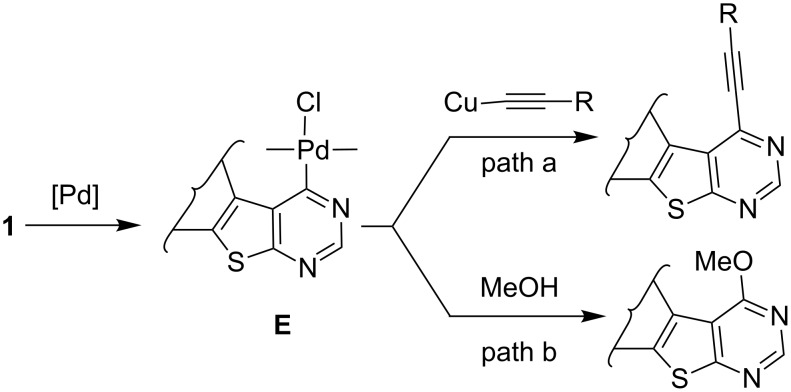
Reactivity of 4-chlorothieno[2,3-*d*]pyrimidines **1** towards terminal alkynes and MeOH under Pd/C–Cu catalysis.

Condensation of resulting amino ester, i.e., ethyl 2-amino-5,6,7,8-tetrahydro-4*H*-cyclohepta[*b*]thiophene-3-carboxylate (**4**) with formamide gave 6,7,8,9-tetrahydro-5*H*-cyclohepta[4,5]thieno[2,3-*d*]pyrimidin-4-one (**5**) which on treatment with POCl_3_ under refluxing conditions provided the desired 4-chloro derivative **1d**. All the terminal alkynes used were commercially available. Initially, we chose to examine the coupling reaction of 4-chloro-5,6,7,8-tetrahydrobenzothieno[2,3-*d*]pyrimidine (**1a**) with phenylacetylene (**2a**) in the presence of 10% Pd/C (0.023 mmol), PPh_3_ (0.17 mmol), CuI (0.04 mmol), and triethylamine (2.67 mmol) in various solvents. The results of this study are summarized in Table 1. The reaction was initially carried out in MeOH for 5 h and the desired product, i.e., 4-(phenylethynyl)-5,6,7,8-tetrahydrobenzothieno[2,3-*d*]pyrimidine (**3a**) was isolated in 67% yield (Table 1, entry 1). The yield of **3a** however was significantly improved when the reaction time was increased to 10 h (Table 1, entry 2). Thus the reaction proceeded well in MeOH to give the expected product via a C–C bond forming reaction (Scheme 3, path a) and no side product as a result of C–O bond formation [11] due to participation of MeOH (Scheme 3, path b) was detected in the reaction mixture. The use of MeOH as a nucleophile in Pd-catalyzed reactions has been well documented in the literature, see for example [12]. Moreover, when the addition of alkyne was omitted without changing the other reaction conditions, compound **1a** reacted with MeOH to give the corresponding ether, 4-methoxy-5,6,7,8-tetrahydrobenzothieno[2,3-*d*]pyrimidine, albeit in low yield. The use of other solvents such as THF, acetonitrile, 1,4-dioxane and DMF (Table 1, entries 3-6) also gave good yields of product but MeOH was found to be superior. Moreover, the reaction temperature (and duration in some cases) was higher, i.e., 80 °C in case of acetonitrile, 1,4-dioxane and DMF compared to 60–65 °C in case of MeOH. While triethylamine was used as a base in all these cases, the use of a secondary amine, e.g., pyrrolidine was also examined. A side product was observed in this case due to the C–N bond forming reaction between **1a** and pyrrolidine and was identified as 4-(*N*-pyrrolidinyl)-5,6,7,8-tetrahydrobenzothieno[2,3-*d*]pyrimidine. The use of another palladium catalyst (PPh_3_)_2_PdCl_2_ was also examined and found to be effective (Table 1, entry 7). However, we preferred to use Pd/C because it is less expensive, easy to handle and separable from the product and has the potential for recyclability, for a review see [13]. To assess the recyclability of the recovered Pd/C-catalyst in the present coupling reaction, the reaction mixture of **1a** and **2a** (Table 1, entry 2) was allowed to cool to room temperature and filtered. The residue was washed with MeOH, acetone, and DCM. After drying under vacuum the recovered catalyst was used directly in the reaction of **1a** with **2a** in the presence of PPh_3_, CuI, and Et_3_N: A conversion of 85% was observed confirming the recyclability of the recovered Pd/C-catalyst.

**Table 1:** Effect of solvent on the coupling reaction of 4-chloro-5,6,7,8-tetrahydrobenzothieno[2,3-*d*]pyrimidine (**1a**) with phenylacetylene (**2a**).^a^

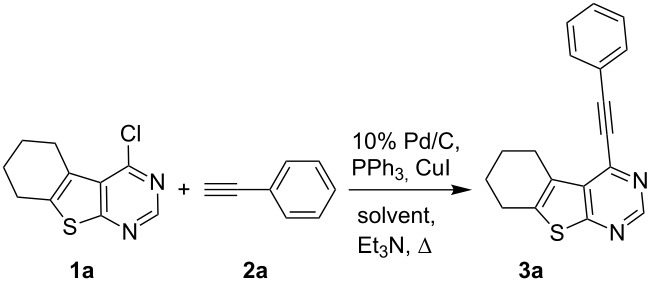			

Entry	Solvent	Time (h)	Yield^b^ (%)

1	MeOH	5	67
2	MeOH	10	90
3	THF	14	80
4	MeCN	12	57 (80^c^)
5	1,4-dioxane	10	59 (80^c^)
6	DMF	10	49 (75^c^)
7	MeOH	10	83^d^

^a^All reactions were carried out by using **1** (0.89 mmol), **2a** (1.33 mmol), 10% Pd/C (0.023 mmol), PPh_3_ (0.17 mmol), CuI (0.04 mmol), and Et_3_N (2.67 mmol) at 60–65 °C under a nitrogen atmosphere. ^b^Isolated yields. ^c^The reaction was carried out at 80 °C. ^d^(PPh_3_)_2_PdCl_2_–CuI was used instead of 10% Pd/C–PPh_3_–CuI.

Having established the optimum reaction conditions for the preparation of **3a** we decided to test the generality and scope of this protocol further. Thus, a variety of commercially available terminal alkynes was employed under the reaction conditions indicated in entry 2 of Table 1 and the results are summarized in Table 2.

**Table 2:** Pd/C-mediated synthesis of 4-alkynylthieno[2,3-*d*]pyrimidine in methanol.^a^

Entry	4-Chlorothieno[2,3-*d*]pyrimidine	R–≡ (R=)	Products^b^	Yield^c^ (%)

1	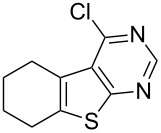 **1a**	–C_6_H_5_ (**2a**)	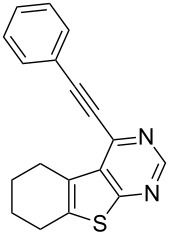 **3a**	90
2	**1a**	–CH_2_OH (**2b)**	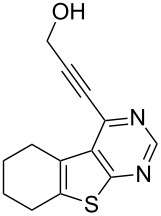 **3b**	88
3	**1a**	–CH_2_CH_2_OH (**2c**)	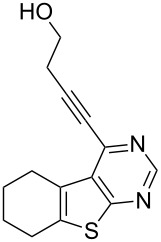 **3c**	85
4	**1a**	–(CH_2_)_2_CH_3_ (**2d**)	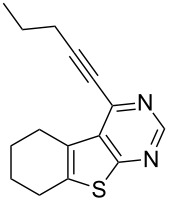 **3d**	75
5	**1a**	–C(CH_3_)_2_OH (**2e**)	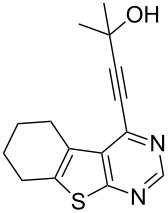 **3e**	77
6	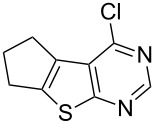 **1b**	–C(CH_3_)_2_OH (**2e**)	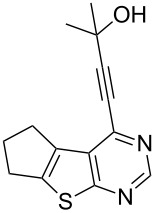 **3f**	80
7	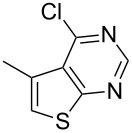 **1c**	–C_6_H_5_ (**2a**)	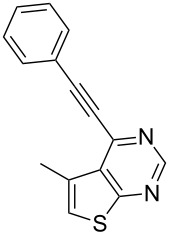 **3g**	90
8	**1c**	–CH_2_OH (**2b**)	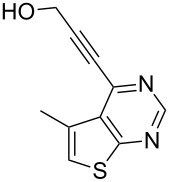 **3h**	85
9	**1c**	–(CH_2_)_2_CH_3_ (**2d**)	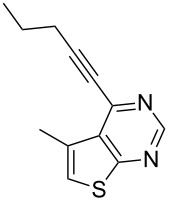 **3i**	79
10	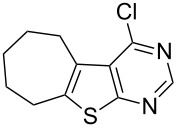 **1d**	–CH_2_OH (**2b**)	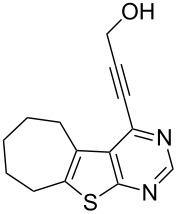 **3j**	87
11	**1d**	–CH_2_CH_2_OH (**2c**)	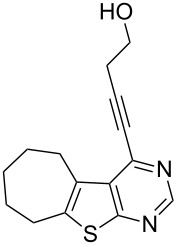 **3k**	88
12	**1d**	–C(CH_3_)_3_ (**2f**)	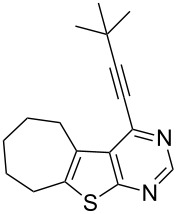 **3l**	85
13	**1d**	–C(CH_3_)_2_OH (**2e**)	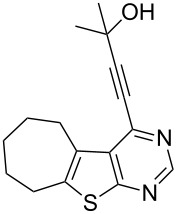 **3m**	85
14	**1d**	–C(CH_2_)_3_CN (**2g**)	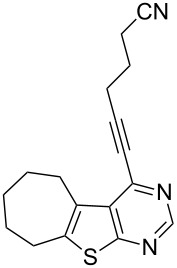 **3n**	80

^a^All the reactions were carried out with **1** (0.89 mmol), **2** (1.33 mmol), 10% Pd/C (0.023 mmol), PPh_3_ (0.17 mmol), CuI (0.04 mmol), and Et_3_N (2.67 mmol) in MeOH (5.0 mL) at 60–65 °C for 10–12 h. ^b^Identified by ^1^H NMR, IR, and MS. ^c^Isolated yields.

As evident from Table 2, the reaction proceeded well with both aliphatic (Table 2, entries 2–6 and 8–14) and aromatic alkynes (Table 2, entry 1 and 7). The chloro compounds containing a five (**1b**), a six (**1a**) or a seven membered cycloalkane ring (**1d**) or without a ring (**1c**) were found to be equally effective under the reaction conditions employed. All the reactions were generally complete within 10 h irrespective of the nature of substituents present in the terminal alkynes **2** to afford the desired products **3a–n** in good to excellent yields.

**Scheme 4:**
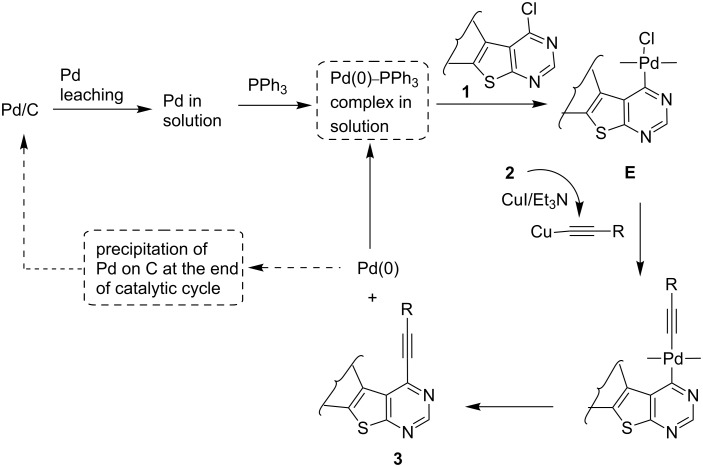
Plausible mechanism of Pd/C-mediated alkynylation of 4-chlorothieno[2,3-*d*]pyrimidines **1**.

A plausible mechanism for the Pd/C–Cu mediated alkynylation of 4-chlorothieno[2,3-*d*]pyrimidines **1** is shown in Scheme 4. The alkynylation proceeds via generation of an active Pd(0) species in situ that undergoes oxidative addition with **1** to give the organo-Pd(II) species **E**. The active Pd(0) species is generated from the minor portion of the bound palladium (Pd/C) via a Pd leaching process in the solution [13]. The leached Pd then becomes an active species by interacting with phosphine ligands. Thus, a soluble Pd(0)–PPh_3_ complex is the active species that actually catalyzes the C–C bond forming reaction in solution. The catalytic cycle therefore works in solution rather than on the surface, and at the end of the reaction, re-precipitation of Pd occurs on the surface of the charcoal. Once generated, the organo-Pd(II) species **E** then facilitates the stepwise formation of C–C bond via (i) trans organometallation with copper acetylide generated in situ from CuI and the terminal alkyne followed by (ii) reductive elimination of Pd(0) to afford 4-alkynylthieno[2,3-*d*]pyrimidine (**3**). A C–O bond forming reaction between **1** and MeOH (Scheme 2, path b) was not observed perhaps due to the higher reactivity of copper acetylide over MeOH (even although present in excess) towards **E**.

**Figure 2:**
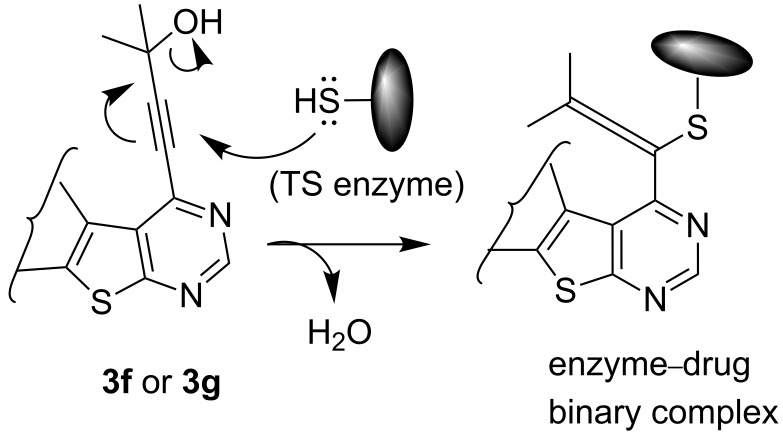
Possible interactions of compounds **3f** and **3g** with TS enzyme.

We have shown that an alkynyl moiety can be introduced efficiently at the C-4 position of a thieno[2,3-*d*]pyrimidine ring via C–C coupling under Pd/C–Cu catalysis. To the best of our knowledge, only two examples of similar coupling have been reported using the system (PPh_3_)_2_PdCl_2_/CuI as catalyst in the presence of Et_3_N [10]. The alkynyl substituent of compound **3** could be utilized for further structural elaboration leading to the functionalized derivatives of thieno[2,3-*d*]pyrimidine preparation which may be difficult to access by other methods. Some of the 4-alkynyl-derivatives synthesized were screened for their cytotoxic activity against chronic myelogenous leukemia (CML) cell line in vitro. The percentage of cell death measured for each compound at two different concentrations, i.e., 10 and 20 µM is shown in Table 3. As evident from Table 3, most of the compounds showed moderate to low activity against CML under the in vitro condition employed. The compounds **3f** and **3g** however, showed reasonable activity when tested at 10 µM which doubled at 20 µM. Comparing the structural features of compounds **3f** and **3g** with the other compounds tested, it may be noted that the presence of CMe_2_OH moiety played an important role in the cytotoxic activities of these compounds. It is known that alkynyl substituted pyrimidine derivatives exhibit their anticancer properties by inhibiting a key enzyme, i.e., thymidylate synthase (TS) which is essential for cellular growth [14]. A possible explanation for observed cytotoxic activities of present series of compounds **3** therefore could be their potential inhibition of TS [15]. The CMe_2_OH group next to the alkynyl moiety may facilitate the binding of the TS enzyme through its sulfhydryl (–SH) moiety with compounds **3f** and **3g** thereby generating the corresponding drug–enzyme allene intermediates (Figure 2). Nevertheless, the present study indicated that 4-alkynylthieno[2,3-*d*]pyrimidine can be used as a template for the identification of novel and potential anticancer agents.

**Table 3:** Cytotoxic activity of 4-alkynylthieno[2,3-*d*]pyrimidines (**3**) against chronic myelogenous leukemia (CML) cell line.

Compound	% of cell death at two concentrations	

	10 µM	20 µM

**3a**	20	26
**3b**	10	24
**3c**	25	26
**3d**	28	36
**3f**	30	54
**3g**	32	60
**3h**	24	26
10% DMSO	5.4	10
Untreated	nil	nil

## Conclusion

In conclusion, the present study demonstrates the first efficient synthesis 4-alkynylthieno[2,3-*d*]pyrimidines in good to excellent yields. The combination of Pd/C–CuI–PPh_3_ proved to be an efficient and selective catalytic system for the C–C bond formation between 4-chlorothieno[2,3-*d*]pyrimidine and terminal alkynes in methanol providing a general and practical method for the preparation of these novel compounds. The reaction proceeds well with both hydrophobic and hydrophilic terminal alkynes without generating any significant side products arising from C–O bond formation or dimerization of the terminal alkynes. The reaction does not involve the use of expensive catalysts or reagents and is easy to perform. Some of the compounds synthesized were tested for cytotoxic activities in vitro. The methodology is amenable to the diversity-oriented synthesis of thieno[2,3-*d*]pyrimidine derivatives of potential pharmacological significance and therefore may find use in organic and medicinal chemistry.

## Supporting Information

Supporting Information features details on experimental procedures and spectral data as well as NMR spectra of compounds **3a**–**n**.

File 1 Experimental procedures and spectral data.

File 2 NMR spectra of compounds **3a**–**n**.
