# Supplementary material for: C–C (alkynylation) vs C–O (ether) bond formation under Pd/C–Cu catalysis: synthesis and pharmacological evaluation of 4-alkynylthieno[2,3-d]pyrimidines
Source: Beilstein J Org Chem. 2011 Mar 21;7:338–45. doi: 10.3762/bjoc.7.44 (PMC3079124; doi:10.3762/bjoc.7.44)
Supplement: File 1 — Experimental procedures and spectral data. [file Beilstein_J_Org_Chem-07-338-s001.pdf]

Supporting Information

for

C–C (alkynylation) vs C–O (ether) bond formation under Pd/C–Cu catalysis: synthesis and pharmacological evaluation of 4-alkynylthieno[2,3-*d*]pyrimidines

Dhilli Rao Gorja[1,2], K. Shiva Kumar[1], K. Mukkanti[2], Manojit Pal*[1]

Address: [1]Institute of Life Sciences, University of Hyderabad Campus, Gachibowli, Hyderabad-500 046, India and [2]Chemistry Division, Institute of Science and Technology, JNT University, Kukatpally, Hyderabad 500072, India.

E-mail: Manojit Pal* - manojitpal@rediffmail.com

*Corresponding author

Experimental procedures and spectral data

Experimental section

General methods: Unless stated otherwise, reactions were performed under a nitrogen atmosphere. Reactions were monitored by thin layer chromatography (TLC) on silica gel plates (60 F254), visualizing with ultraviolet light or iodine spray. Flash chromatography was performed on silica gel (100–200 mesh) using distilled hexane, ethyl acetate, dichloromethane. ^{1}H NMR and ^{13}C NMR spectra were determined in $CDCl_3$ solution on a Varian 400 spectrometer at 400 MHz and 100 MHz, respectively. Proton chemical shifts (δ) are relative to tetramethylsilane (TMS, δ = 0.00) as internal standard and expressed in ppm. Spin multiplicities are given as s (singlet), d (doublet), t (triplet) and m (multiplet) as well as b (broad). Coupling constants (*J*) are given in Hz. Infrared spectra were recorded on a JASCO FT-IR-4200 spectrometer. Mass spectra were obtained on an AGILENT-6430 LC-MS/MS-Quadrupole spectrometer. Melting points are uncorrected.

General procedure for the preparation of compound 3: A mixture of the 4-chlorothieno[2,3-*d*]pyrimidine (**1**) (0.89 mmol), 10% Pd/C (0.023 mmol), PPh_3 (0.17 mmol), CuI (0.04 mmol), and triethylamine (2.67 mmol) in methanol (5 mL) was stirred at 25–30 °C for 30 min under a nitrogen atmosphere. The acetylenic compound (**2**) (1.33 mmol) was added, and the mixture was initially stirred at room temperature for 1 h and then at 60–65 °C for 10–12 h. After completion of the reaction, the mixture was cooled to room temperature, diluted with EtOAc (50 mL), and filtered through Celite. The organic solution was washed with water (3 × 30 mL), dried over anhydrous Na_2SO_4, and concentrated. The crude residue was purified by column chromatography on silica gel using hexane/ethyl acetate to afford the desired product.

5,6,7,8 tetrahydrobenzo-4-(phenylethynyl)thieno[2,3-d]pyrimidine (3a)

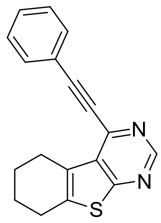

Yellow solid, R_f (25% ethyl acetate/*n*-hexane) 0.45; mp 133–135 °C; ^{1}H NMR (400 MHz, $CDCl_3$) δ 8.94 (s, 1H), 7.65–7.63 (m, 2H), 7.45–7.40 (m, 3H), 3.25 (m, 2H), 2.92 (m, 2H), 1.99–1.96 (m, 4H); ^{13}C NMR (100 MHz, $CDCl_3$) δ 175.9, 172.2, 168.7, 152.0, 142.2, 139.1, 131.9, 130.6, 129.8, 128.5, 127.3, 121.4, 96.2, 86.9, 25.9, 25.3, 22.5, 22.2; IR (KBr) $ν_{max}$ 2924, 2212, 1423, 1436 cm^{-1}; *m*/*z* (CI) 291 ([M + 1], 100%).

3-(5,6,7,8 tetrahydrobenzothieno[2,3-d]pyrimidin-4-yl)prop-2-yn-1-ol (3b)

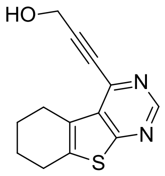

Brown oil; R_f (35% ethyl acetate/*n*-hexane) 0.54; ^{1}H NMR (400 MHz, $CDCl_3$) δ 8.92 (s, 1H), 4.61 (s, 2H), 4.35 (s, 1H), 3.01-3.08 (m, 2H), 2.90-2.88 (m, 2H), 1.93-1.91 (m, 4H); ^{13}C NMR (100 MHz, $CDCl_3$) δ 168.9, 151.7, 141.4, 139.5, 130.6, 127.2, 95.7, 82.5, 51.1, 26.0, 25.1, 22.5, 22.1; IR (KBr) $ν_{max}$ 2932, 2228, 1523, 1318 cm^{-1}; m/z (CI) 245 (M+1, 100%).

4-(5,6,7,8 tetrahydrobenzothieno[2,3-d]pyrimidin-4-yl)but-3-yn-1-ol (3c)

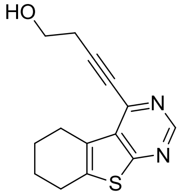

Yellow semi-solid; R_f (35% ethyl acetate/*n*-hexane) 0.56; ^{1}H NMR (400 MHz, $CDCl_3$) δ 8.86 (s, 1H), 3.92 (t, *J* = 6.0 Hz, 2H), 3.75 (t, *J* = 6.0 Hz, 2H), 3.08–3.00 (m, 2H), 2.98–2.95 (m, 2H), 2.83–2.80 (m, 2H), 2.55–2.52 (m, 2H); ^{13}C NMR (100 MHz, $CDCl_3$) δ 151.8, 142.2, 139.0, 132.1, 130.9, 127.3, 96.5, 79.9, 60.3, 25.9, 25.1, 24.2, 22.5, 22.1; IR (KBr) $ν_{max}$ 2937, 2228, 1530, 1063 cm^{-1}; *m*/*z* (CI) 259 ([M + 1], 100%).

4-(pent-1-ynyl)-5,6,7,8 tetrahydrobenzothieno[2,3-d]pyrimidine (3d)

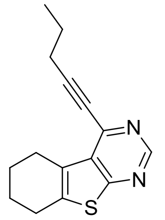

Light brown oil; R_f (25% ethyl acetate/*n*-hexane) 0.52; ^{1}H NMR (400 MHz, $CDCl_3$) δ 8.86 (s, 1H), 3.10–3.08 (m, 2H), 2.98–2.96 (m, 2H), 2.56–2.53 (m, 2H), 1.93–1.91 (m, 4H), 1.70–1.68 (m, 2H), 1.07 (m, 3H); ^{13}C NMR (100 MHz, $CDCl_3$) δ 168.5, 151.9, 142.8, 138.3, 130.5, 127.3, 99.1, 78.8, 25.8, 25.2, 22.5, 22.1, 21.6, 21.4, 13.6; IR (KBr) ν_{max} 2925, 2232, 1531, 1050 cm^{-1}; *m*/*z* (CI) 257 ([M + 1], 100%).

2-methyl-4-(5,6,7,8 tetrahydrobenzothieno[2,3-d]pyrimidin-4-yl)but-3-yn-2-ol (3e)

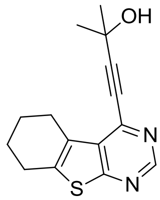

Light brown oil; R_f (30% ethyl acetate/*n*-hexane) 0.52; ^{1}H NMR (400 MHz, $CDCl_3$) δ 8.86 (s, 1H), 4.07 (bs, 1H), 3.09-3.12 (m, 2H), 2.86-2.87 (m, 2H), 1.92- 1.89 (m, 4H), 1.55 (s, 3H), 1.39 (s, 3H); ^{13}C NMR (100 MHz, $CDCl_3$) δ 156.8, 151, 142.8, 138.1, 130.4, 127.2, 106.1, 77.4, 53.4, 29.9, 29.4, 22.7, 22.6, 22.3, 22.1; IR (KBr) ν_{max} 2923, 2225, 1631, 1363 cm^{-1}; *m*/*z* (CI) 273 ([M + 1], 100%).

2-methyl-4-(6,7-dihydro-5H-cyclopenta[4,5]thieno[2,3-d]pyrimidin-4-yl)but-3-yn-2-ol (3f)

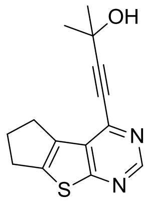

Light brown oil; R_f (30% ethyl acetate/*n*-hexane) 0.52; ^{1}H NMR (400 MHz, $CDCl_3$) δ 8.89 (s, 1H), 5.10 (bs, 1H), 2.90–2.93 (m, 2H), 2.56–2.53 (m, 2H), 1.93–1.91 (m, 2H), 1.61 (s, 3H), 1.55 (s, 3H); ^{13}C NMR (100 MHz, $CDCl_3$) δ 168.8, 151.9, 149.0, 143.7, 141.2, 139.3, 101.2, 77.2, 65.4, 31.3, 30.8, 30.7, 25.9, 25.3; IR (KBr) v_{max} 3379 3352, 2976, 2222, 1727 cm^{-1}; *m*/*z* (CI) 259 ([M + 1], 100%).

5-methyl-4-(phenylethynyl)thieno[2,3-d]pyrimidine (3g)

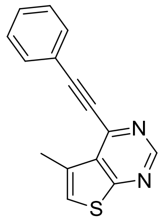

Pale brown solid, R_f (25% ethyl acetate/*n*-hexane) 0.45; mp 122–125 °C; ^{1}H NMR (400 MHz, $CDCl_3$) δ 9.03 (s, 1H), 7.67–7.65 (m, 2H), 7.46–7.42 (m, 3H), 7.26 (s, 1H), 2.82 (s, 3H); ^{13}C NMR (100 MHz, $CDCl_3$) δ 170.1, 152.9, 144.4, 132.0, 130.6 (2C), 130.0 (2C), 129.8, 128.6, 123.2, 121.3, 97.1, 86.8, 16.8; IR (KBr) v_{max} 3101, 2922, 2211, 1522 cm^{-1}; *m*/*z* (CI) 251 ([M + 1], 100%).

3-(5-methylthieno[2,3-d]pyrimidin-4-yl)prop-2-yn-1-ol (3h)

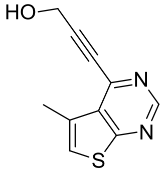

Yellow solid, R_f (35% ethyl acetate/*n*-hexane) 0.58; mp 118–120 °C; ^{1}H NMR (400 MHz, $CDCl_3$) δ 8.99 (s, 1H), 7.26 (s, 1H), 4.63 (s, 2H), 2.70 (s, 3H), 2.39 (bs, 1H); ^{13}C NMR (100 MHz, $CDCl_3$) δ 170.2, 152.4, 143.6, 130.5, 129.8, 123.4, 96.5, 82.3, 51.2, 16.5; IR (KBr) v_{max} 2924, 2229, 1731, 1363 cm^{-1}; *m*/*z* (CI) 205 ([M + 1], 100%).

5-methyl-4-(pent-1-ynyl)thieno[2,3-d]pyrimidine (3i)

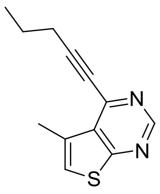

Light brown oil; R_f (25% ethyl acetate/*n*-hexane) 0.58; ^{1}H NMR (400 MHz, $CDCl_3$) δ 8.96 (s, 1H), 7.26 (s, 1H), 2.69 (s, 3H), 2.56–2.53 (m, 2H), 1.76–1.74 (m, 2H), 1.11–1.07 (m, 3H); ^{13}C NMR (100 MHz, $CDCl_3$) δ 169.9, 152.8, 145.0, 130.7, 129.8, 122.5, 100.2, 78.7, 21.7, 21.4, 14.0, 13.7; IR (KBr) v_{max} 2968, 2230, 1518, 1155 cm^{-1}; *m*/*z* (CI) 217 ([M + 1], 100%).

3-(6,7,8,9)-tetrahydro-5*H*-cyclohepta[4,5]thieno[2,3-*d*]pyrimidin-4-yl)prop-2-yn-1-ol (3j)

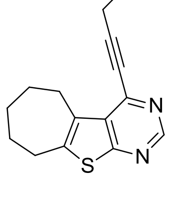

Light brown oil; R_f (35% ethyl acetate/*n*-hexane) 0.50; ^{1}H NMR (400 MHz, $CDCl_3$) δ 8.89 (s, 1H), 4.62 (s, 2H), 3.40–3.38 (m, 2H), 2.98–2.96 (m, 2H), 1.94–1.92 (m, 2H), 1.81–1.75 (m, 4H);

^{13}C NMR (100 MHz, $CDCl_3$) δ 167.7, 151.1, 143.3, 141.4, 132.3, 130.6, 96.6, 82.3, 51.0, 29.9, 28.6, 27.1, 26.7, 26.3; IR (KBr) $ν_{max}$ 3230, 3072, 2926, 2856, 2228, 1277 cm^{-1}; *m/z* (CI) 259 ([M + 1], 100%).

3-(6,7,8,9-tetrahydro-5*H*-cyclohepta[4,5]thieno[2,3-*d*]pyrimidin-4-yl)but-3-yn-1-ol (3k)

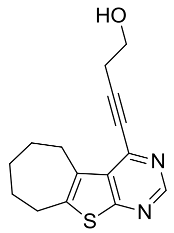

Light brown oil; R_f (35% ethyl acetate/*n*-hexane) 0.54; ^{1}H NMR (400 MHz, $CDCl_3$) δ 8.87 (s, 1H), 3.92–3.90 (m, 2H), 3.76–3.75 (m, 1H), 3.43–3.40 (m, 2H), 2.98–2.95 (m, 2H), 2.83 (t, *J* = 5.6 Hz, 2H), 1.95–1.93 (m, 2H), 1.79–1.75 (m, 4H); ^{13}C NMR (100 MHz, $CDCl_3$) δ 167.7, 151.2, 142.2, 132.6, 131.9, 130.7, 97.9, 79.6, 60.2, 30.2, 30.1, 27.2, 27.0, 26.6, 23.6; IR (KBr) $ν_{max}$ 3283, 3079, 2927, 2225, 1728, 1439 cm^{-1}; *m/z* (CI) 272.9 ([M + 1], 100%).

4-(3,3-dimethylbut-1-ynyl)-6,7,8.9-tetrahydro-5*H*-cyclohepta[4,5]thieno[2,3-*d*]pyrimidine (3l)

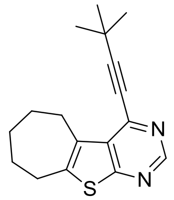

Pale yellow oil; R_f (30 % ethyl acetate/*n*-hexane) 0.54; ^{1}H NMR (400 MHz, $CDCl_3$) δ 8.87 (s, 1H), 3.47–3.44 (m, 2H), 2.98–2.95 (m, 2H), 1.96–1.94 (m, 2H), 1.79–1.72 (m, 4H), 1.40 (s, 9H); ^{13}C NMR (100 MHz, $CDCl_3$) δ 167.5, 151.6, 143.0, 142.6, 132.7, 130.8, 107.5, 77.4, 32.1 (3C), 30.2, 30.1, 28.4, 27.2, 27.1, 26.5; IR (KBr) $ν_{max}$ 2923, 2858, 2220, 1509, 1133 cm^{-1}; *m/z* (CI) 285 ([M + 1], 100%).

2-methyl-4-(6,7,8,9-tetrahydro-5*H*-cyclohepta[4,5]thieno[2,3-*d*]pyrimidin-4-yl)but-3-yn-2-ol (3m)

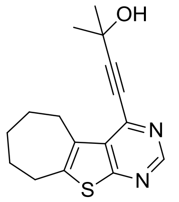

Pale yellow oil; R_f (35% ethyl acetate/*n*-hexane) 0.58; ^{1}H NMR (400 MHz, $CDCl_3$) δ 8.90 (s, 1H), 3.43–3.40 (m, 2H), 2.98–2.96 (m, 2H), 2.36 (bs, 1H), 1.95–1.94 (m, 2H), 1.78–1.73 (m, 4H), 1.68 (s, 6H); ^{13}C NMR (100 MHz, $CDCl_3$) δ 167.7, 151.4, 143.4, 141.9, 132.5, 130.9, 102.7, 79.5, 65.3, 31.9, 31.0, 30.7, 30.1, 27.3, 27.0, 26.5; IR (KBr) ν_{max} 3401, 3052, 2932, 2220, 1519, 1166 cm^{-1}; *m*/*z* (CI) 287 ([M + 1], 100%).

6-(6,7,8,9-tetrahydro-5*H*-cyclohepta[4,5]thieno[2,3-d]pyrimidin-4-yl)hex-5-ynenitrile (3n)

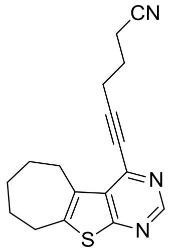

Pale yellow oil; R_f (30% ethyl acetate/*n*-hexane) 0.52; ^{1}H NMR (400 MHz, $CDCl_3$) δ 8.88 (s, 1H), 3.41–3.38 (m, 2H), 2.99–2.96 (m, 2H), 2.79–2.76 (m, 2H), 2.61–2.58 (m, 2H), 2.52–2.46 (m, 2H), 2.09–2.04 (m, 2H), 1.96–1.91 (m, 4H); ^{13}C NMR (100 MHz, $CDCl_3$) δ 151.4, 143.2, 141.9, 132.2, 130.8, 118.6, 96.4, 80.1, 75.0, 29.7, 27.2, 26.9, 26.8, 26.5, 24.0, 23.8, 18.6; IR (KBr) ν_{max} 2926, 2850, 2229, 1515 cm^{-1}; *m*/*z* (CI) 296 ([M + 1], 100%).

Cytotoxicity assay

Some of the compounds synthesized were screened for their cytotoxic activity against chronic myelogenous leukemia (CML). Test compounds were dissolved in DMSO and were diluted appropriately with culture media DMEM (Dulbecco's Modified Eagle's Medium) prior to treatment of cells.

The assay was carried out in a 96 well plate. The cell lines were incubated for 24 h in 100 µL medium. After sufficient growth was observed, different concentrations of the samples were added into each well respectively, with 10% DMSO as a control. After 24 h of incubation the plate was centrifuged and the supernatant was discarded. Then 100 µL of 0.4 mg/mL MTT or tetrazolium salt [3-(4,5-dimethylthiazol-2-yl)-2,5-diphenyltetrazolium bromide] in PBS (phosphate-buffered saline) was added to the settled cell lines (after centrifugation). After 4 h the plate was again centrifuged and the supernatant was discarded. DMSO and 100 µL of 1:1 DMSO–ethanol were added to the settled cell lines and the plate was measured at OD_{595}. The data obtained were used to assess the percentage of cell death.

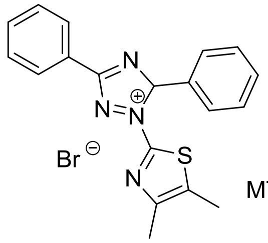

MTT salt
