# Supplementary material for: C–C (alkynylation) vs C–O (ether) bond formation under Pd/C–Cu catalysis: synthesis and pharmacological evaluation of 4-alkynylthieno[2,3-d]pyrimidines
Source: Beilstein J Org Chem. 2011 Mar 21;7:338–45. doi: 10.3762/bjoc.7.44 (PMC3079124; doi:10.3762/bjoc.7.44)

Supporting Information

for

C–C (alkynylation) vs C–O (ether) bond formation under Pd/C–Cu catalysis: synthesis and pharmacological evaluation of 4-alkynylthieno[2,3-*d*]pyrimidines

Dhilli Rao Gorja[1,2], K. Shiva Kumar[1], K. Mukkanti[2], Manojit Pal*[1]

Address: [1]Institute of Life Sciences, University of Hyderabad Campus, Gachibowli, Hyderabad-500 046, India and [2]Chemistry Division, Institute of Science and Technology, JNT University, Kukatpally, Hyderabad 500072, India.

E-mail: Manojit Pal* - manojitpal@rediffmail.com

*Corresponding author

NMR spectra of compounds 3a–n

3a ^{1}H NMR (400 MHz, $CDCl_3$)

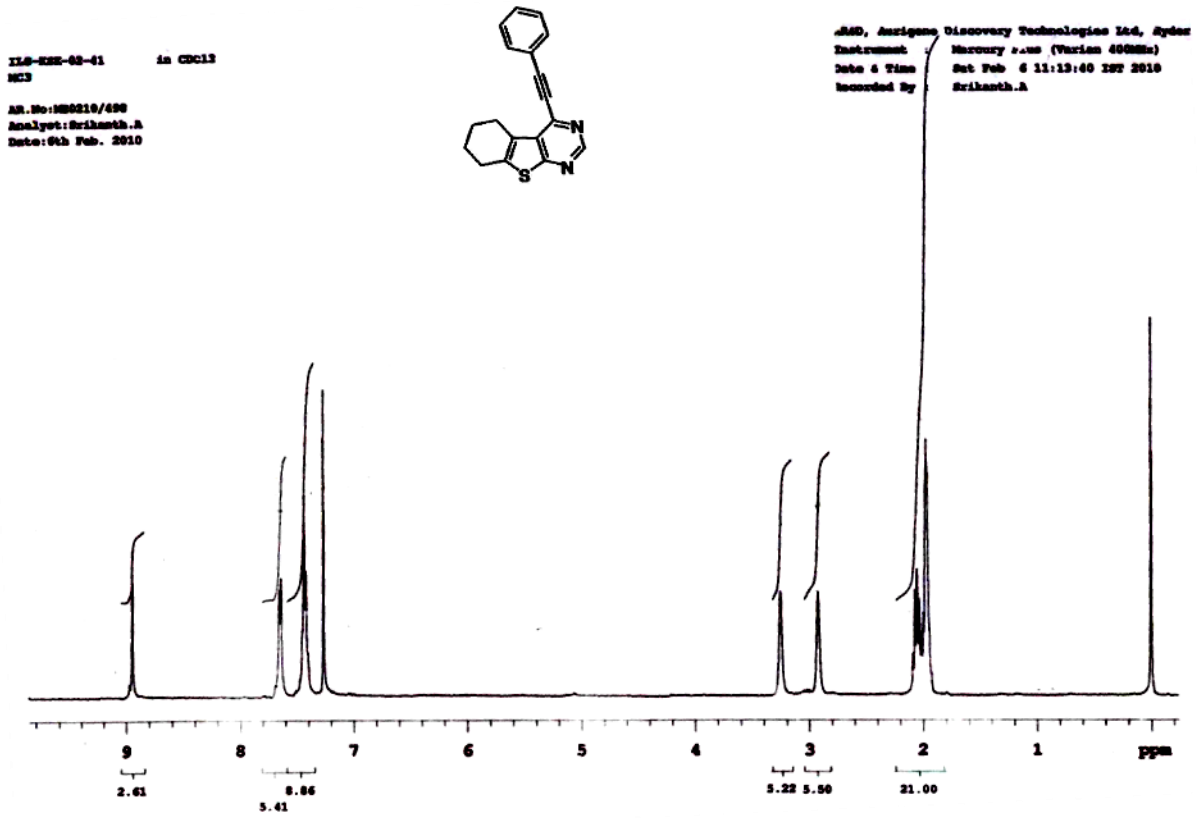

3a ^{13}C NMR (100 MHz, $CDCl_3$)

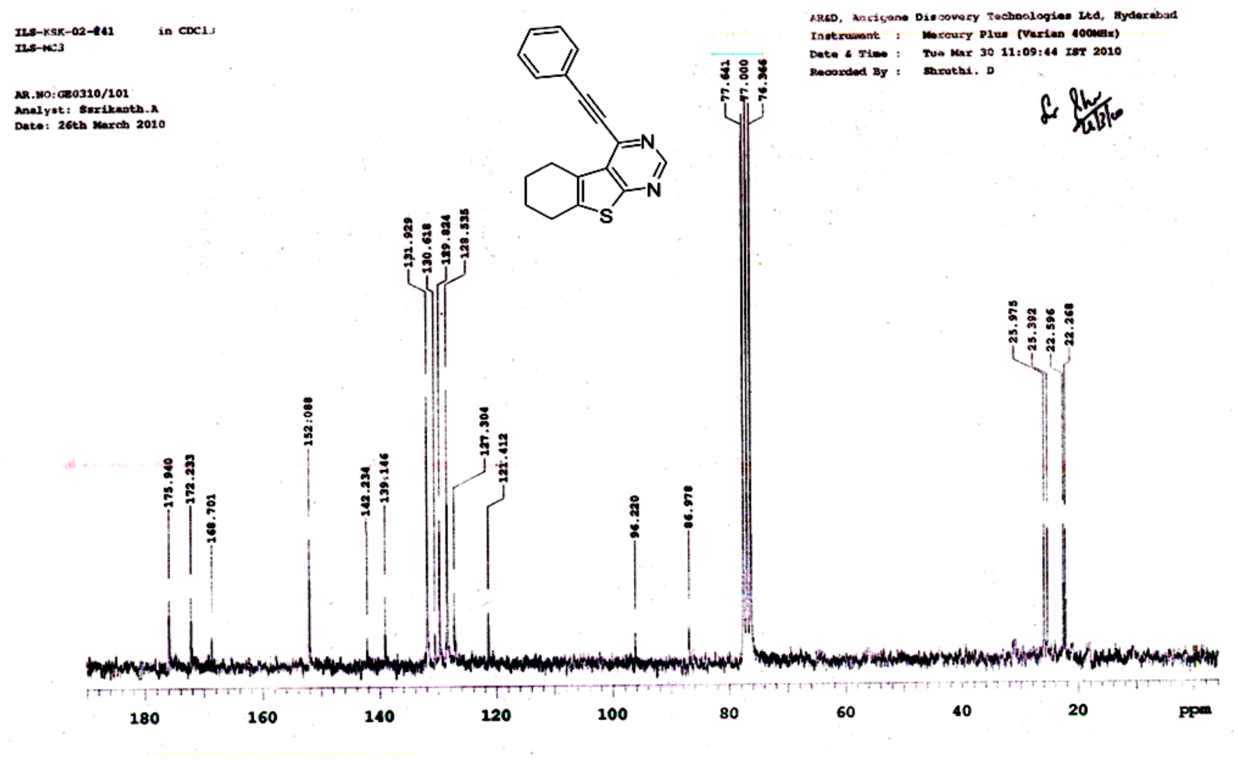

3b ^{1}H NMR (400 MHz, $CDCl_3$)

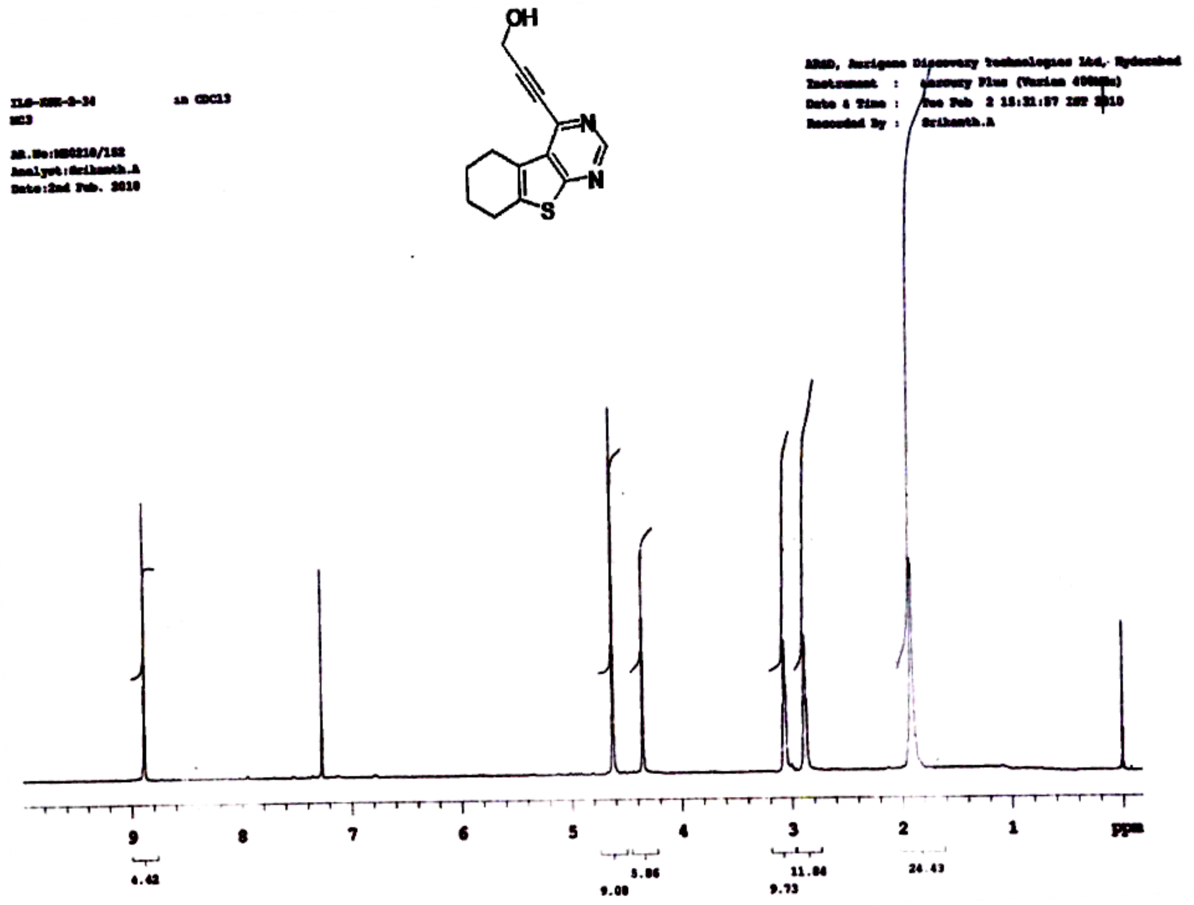

3b ^{13}C NMR (100 MHz, $CDCl_3$)

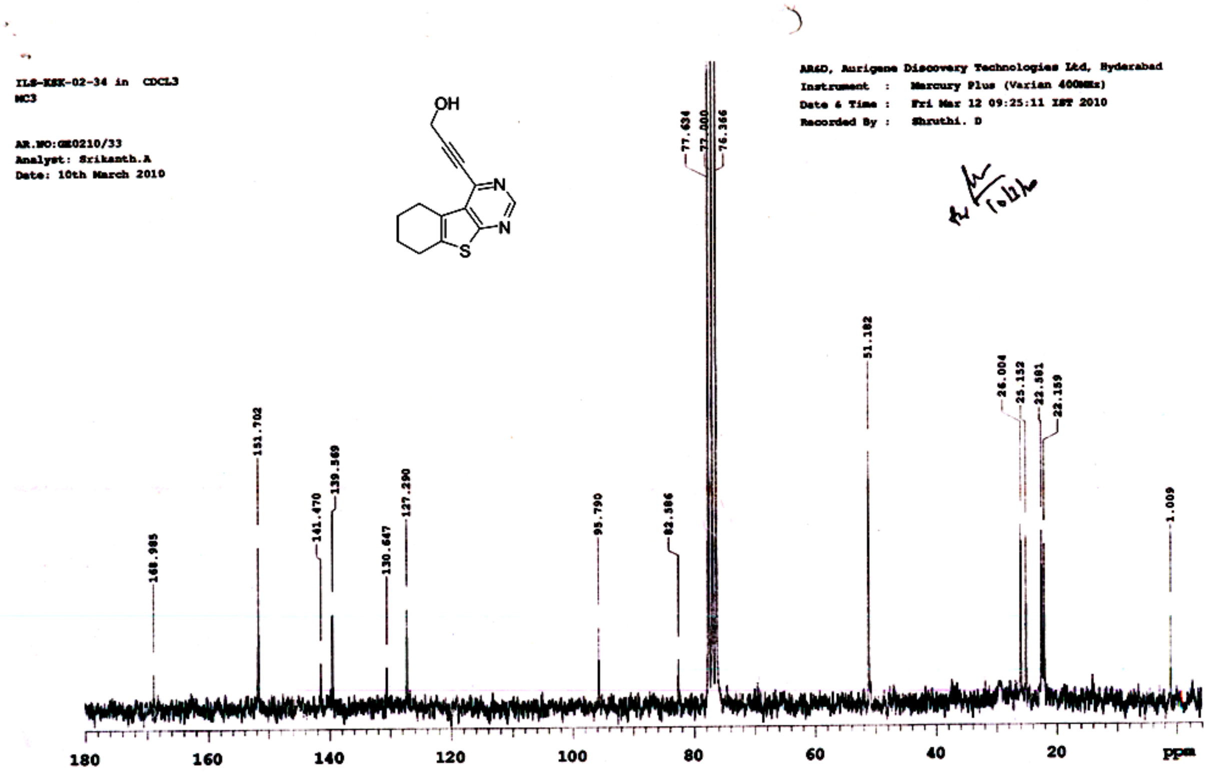

3c ^{1}H NMR (400 MHz, $CDCl_3$)

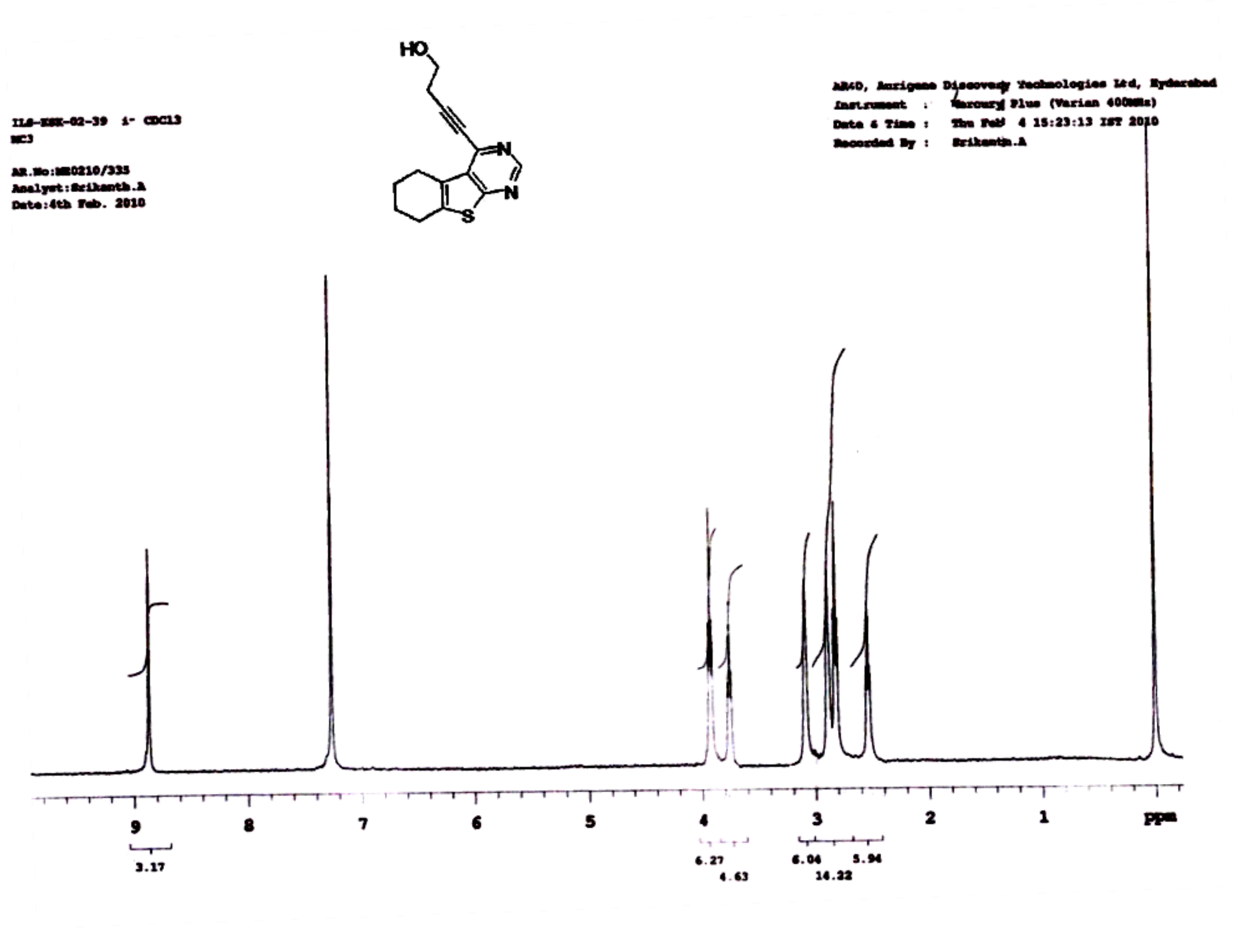

3c ^{13}C NMR (100 MHz, $CDCl_3$)

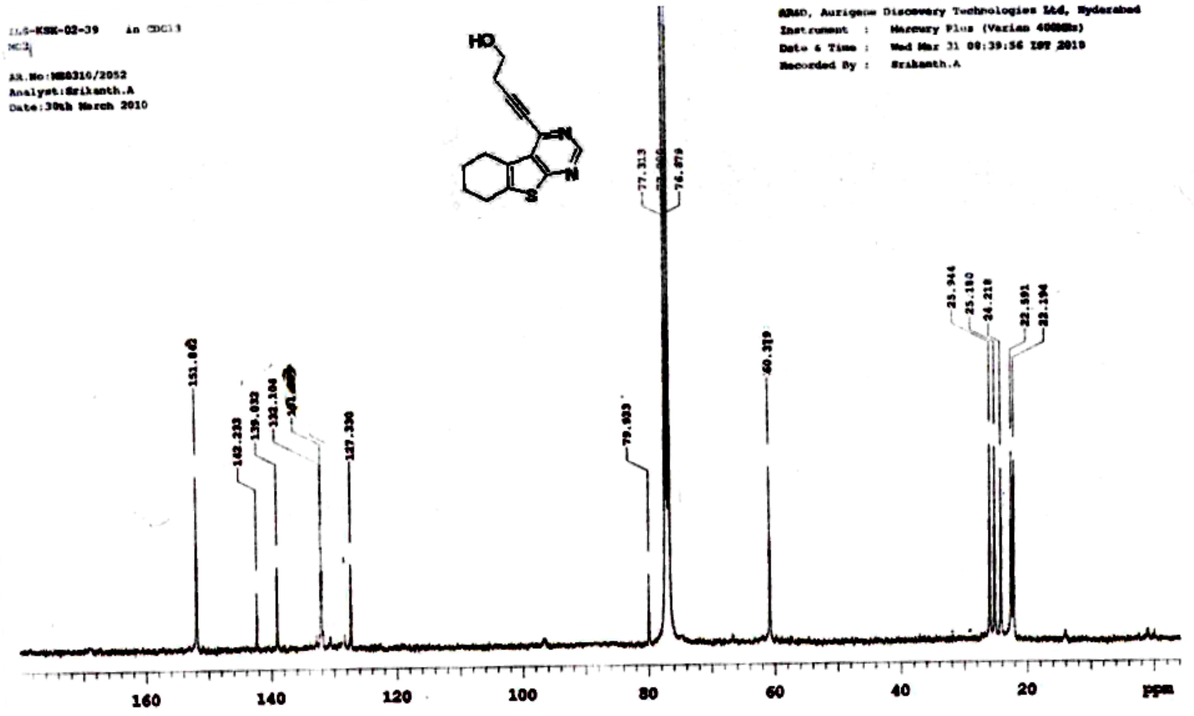

3d ^{1}H NMR (400 MHz, $CDCl_3$)

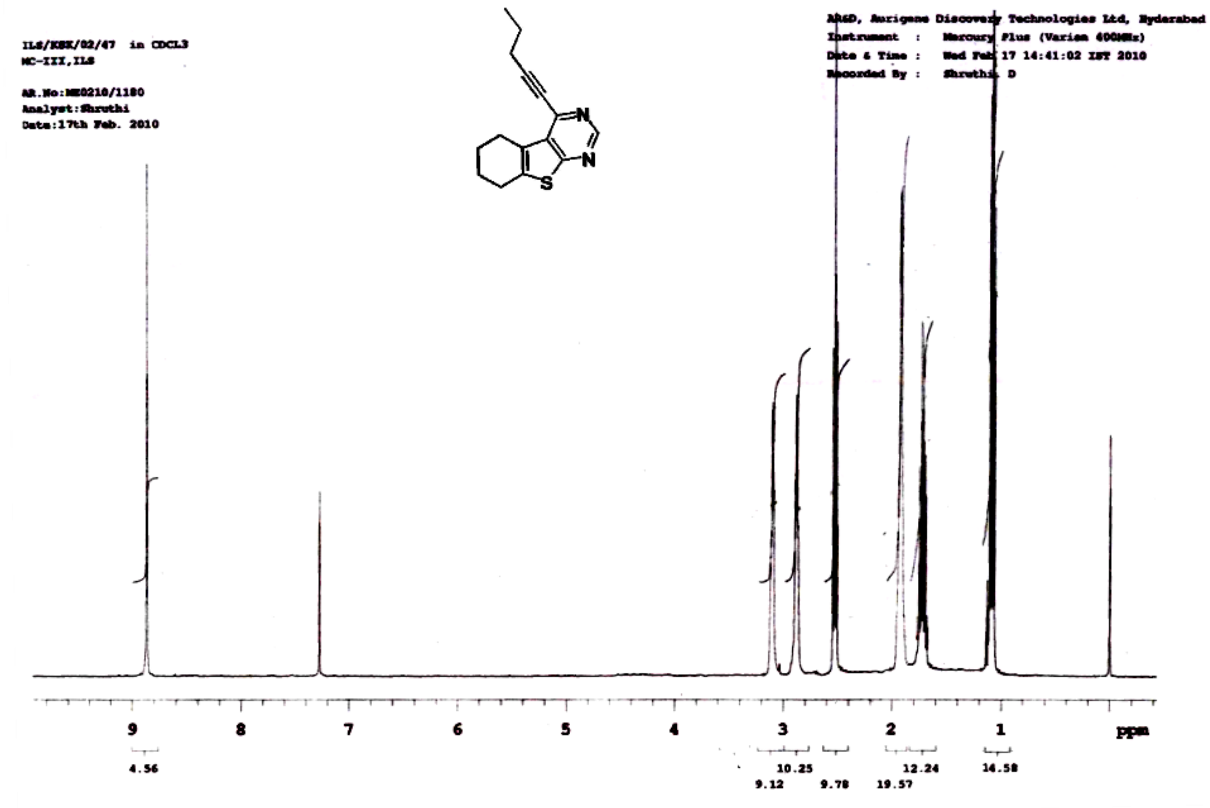

3d ^{13}C NMR (100 MHz, $CDCl_3$)

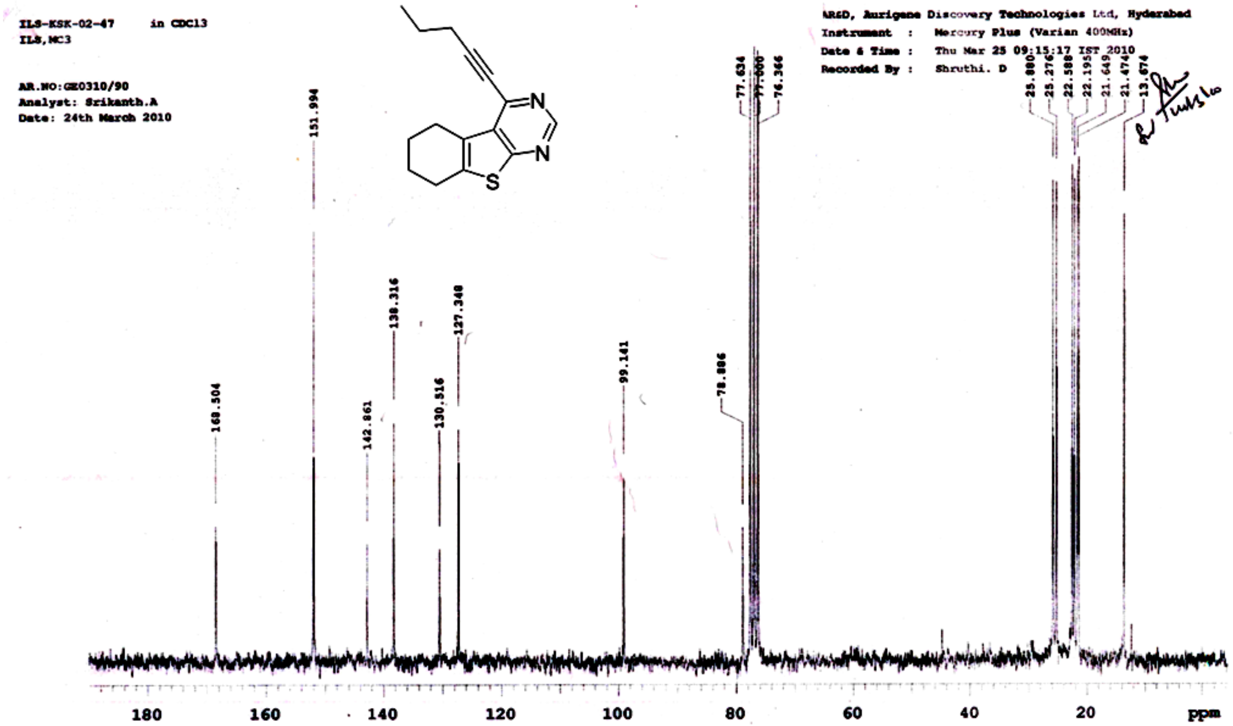

3e ^{1}H NMR (400 MHz, $CDCl_3$)

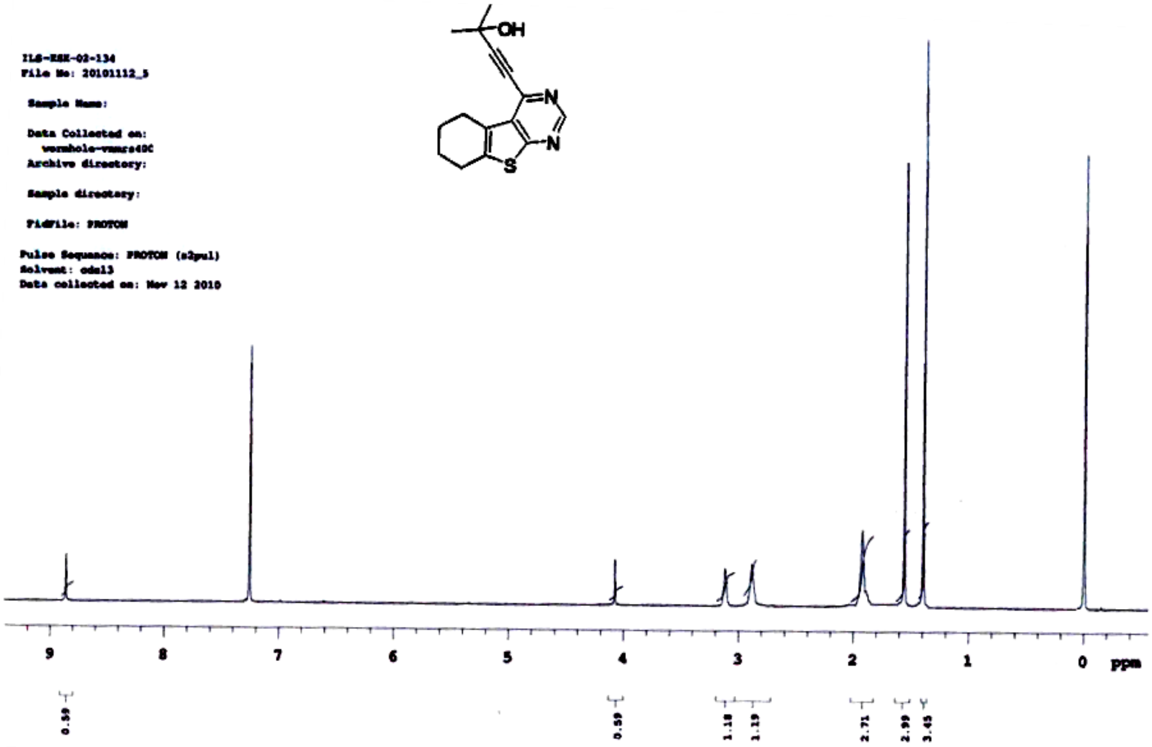

3e ^{13}C NMR (100 MHz, $CDCl_3$)

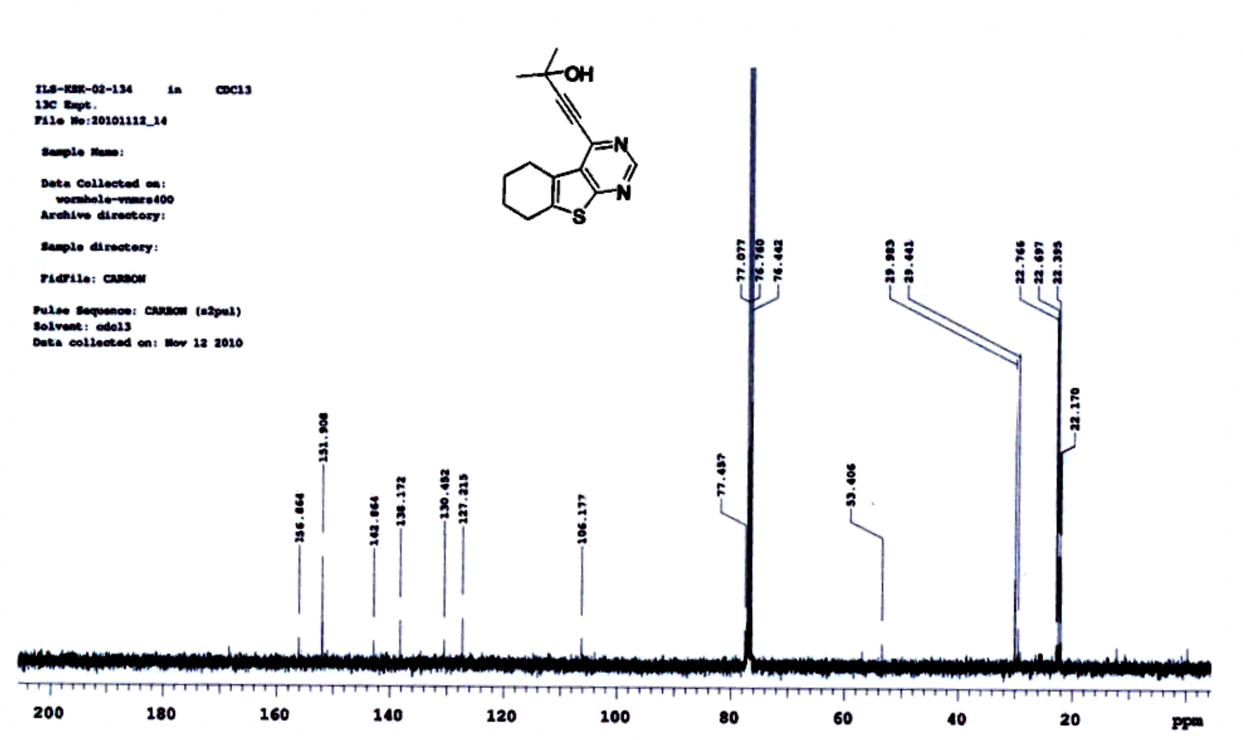

3f ^{1}H NMR (400 MHz, $CDCl_3$)

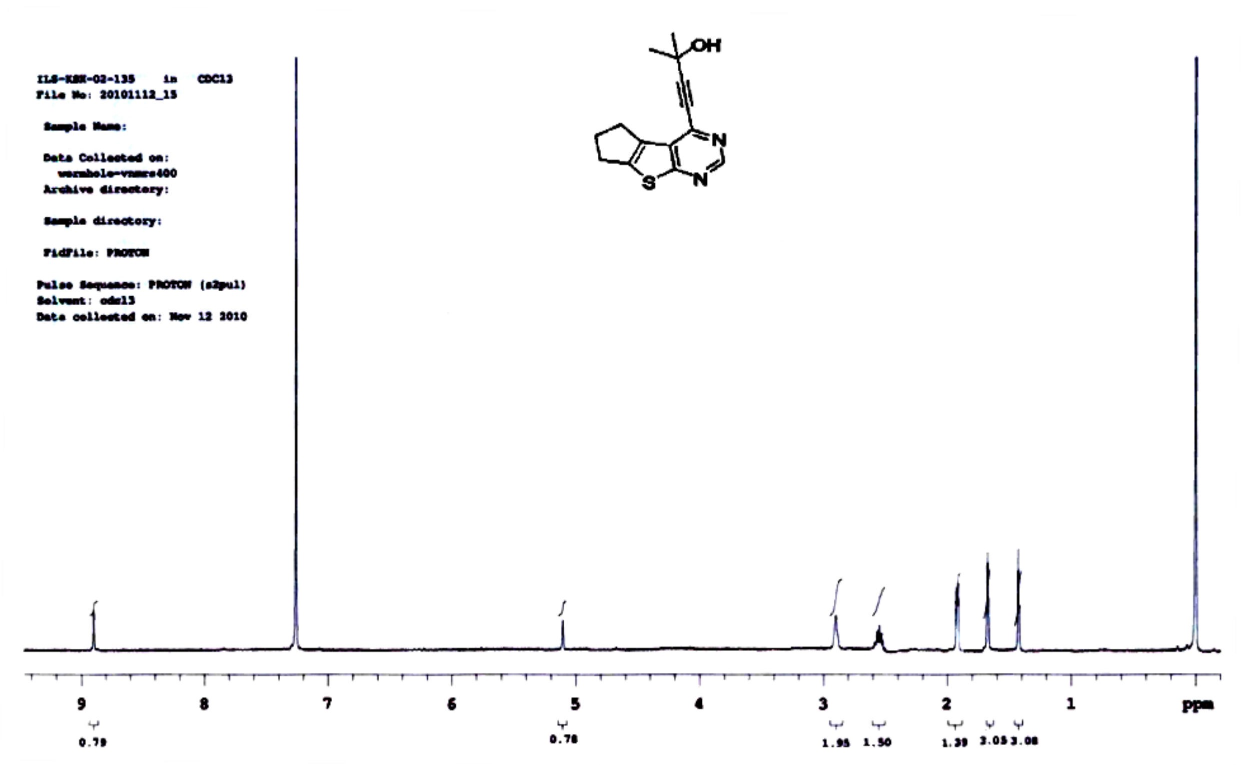

3f ^{13}C NMR (100 MHz, $CDCl_3$)

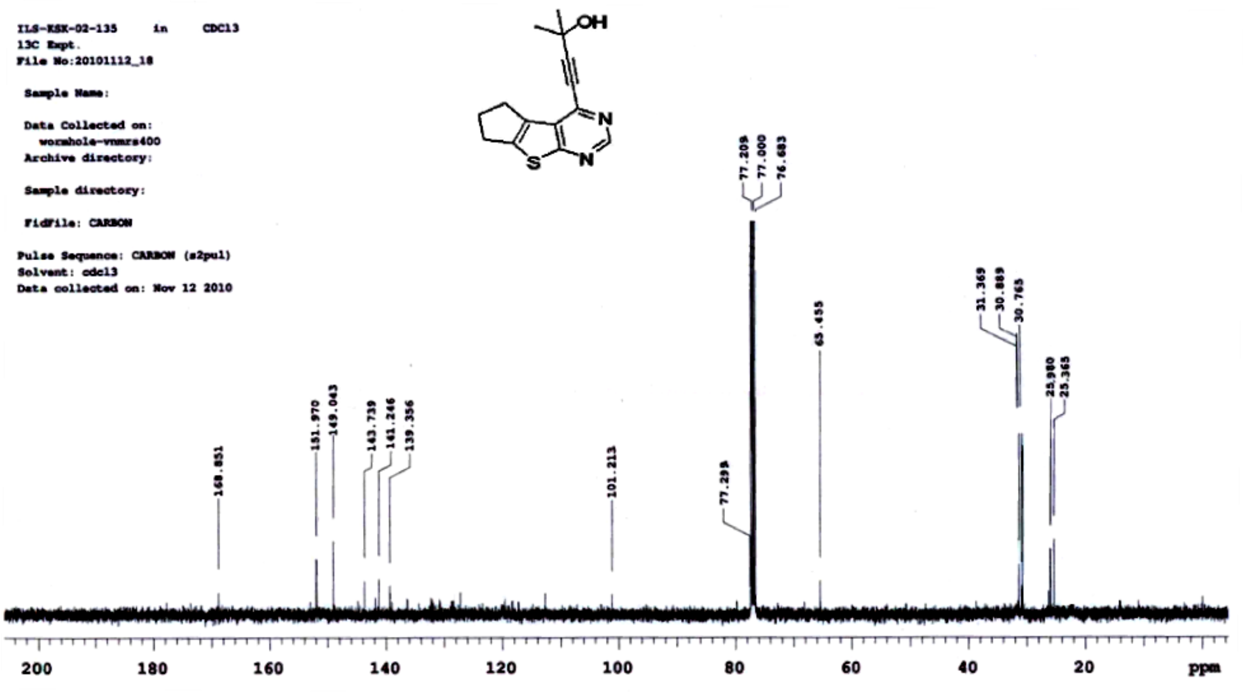

3g ^{1}H NMR (400 MHz, $CDCl_3$)

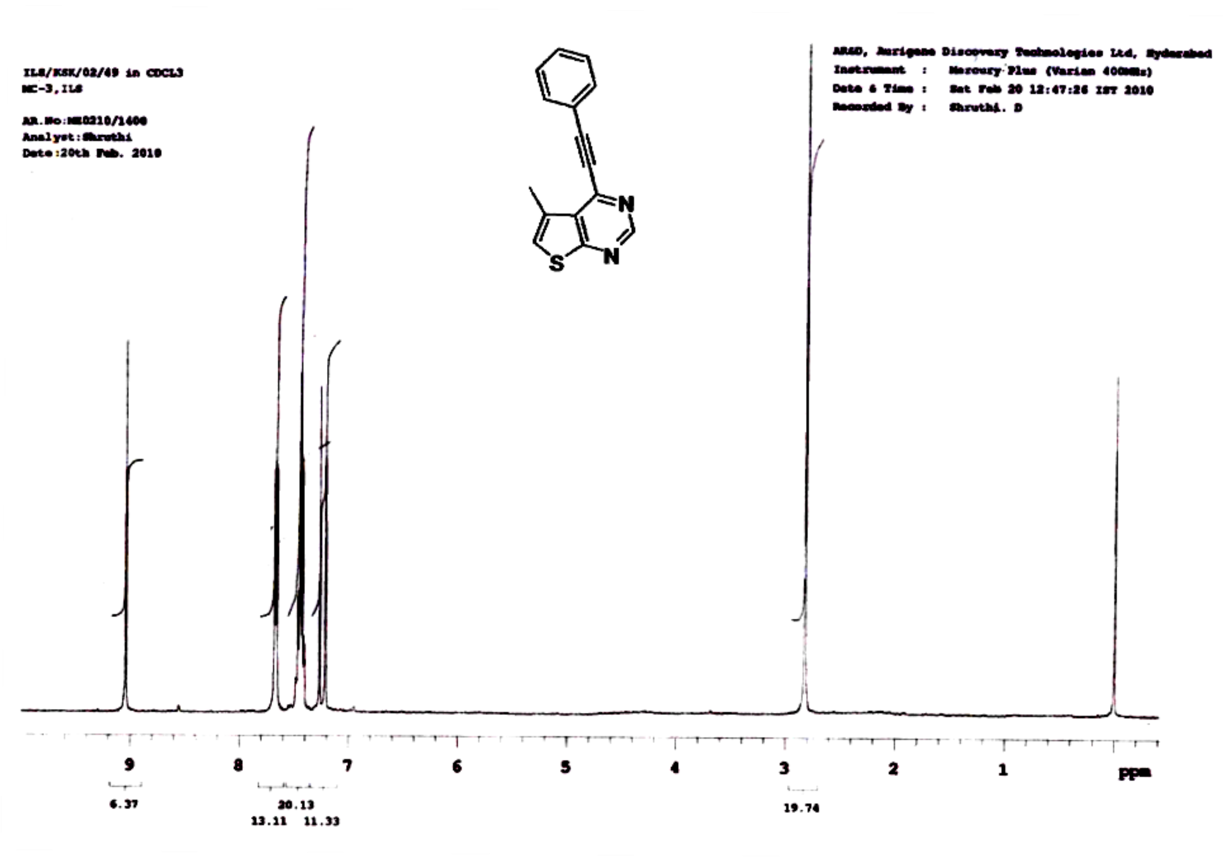

3g ^{13}C NMR (100 MHz, $CDCl_3$)

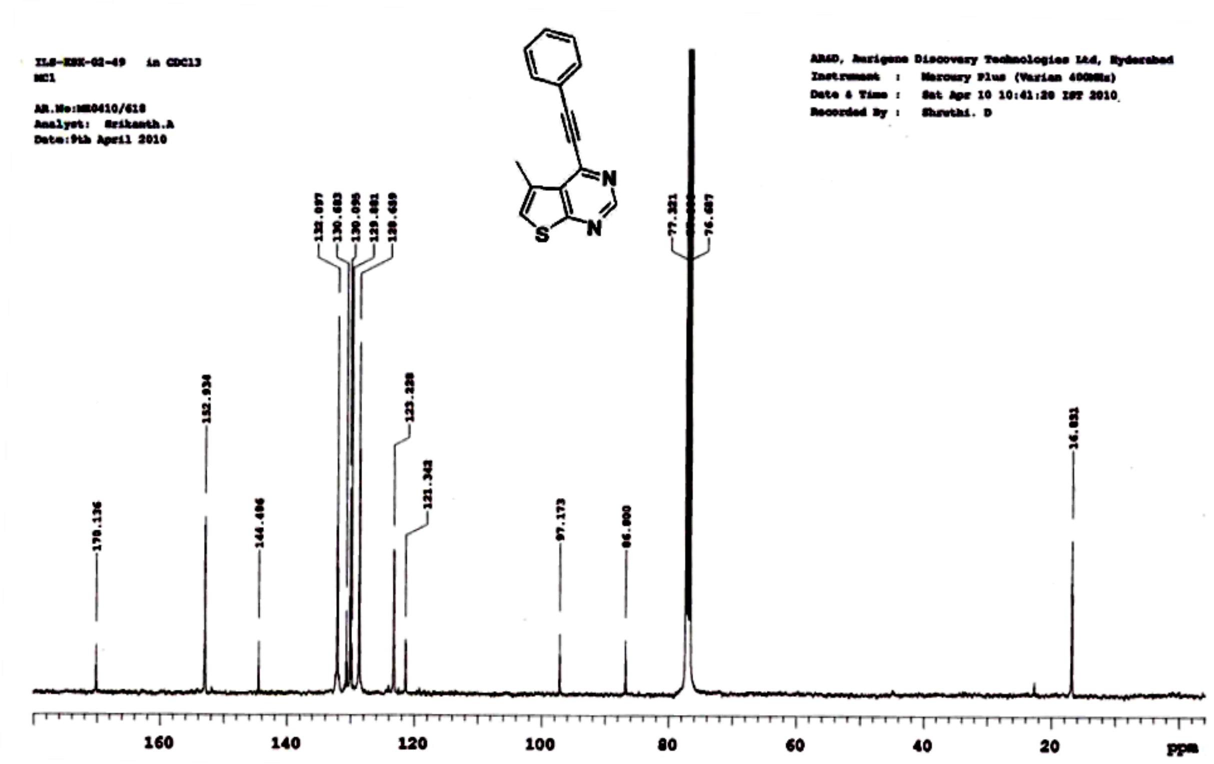

3h ^{1}H NMR (400 MHz, $CDCl_3$)

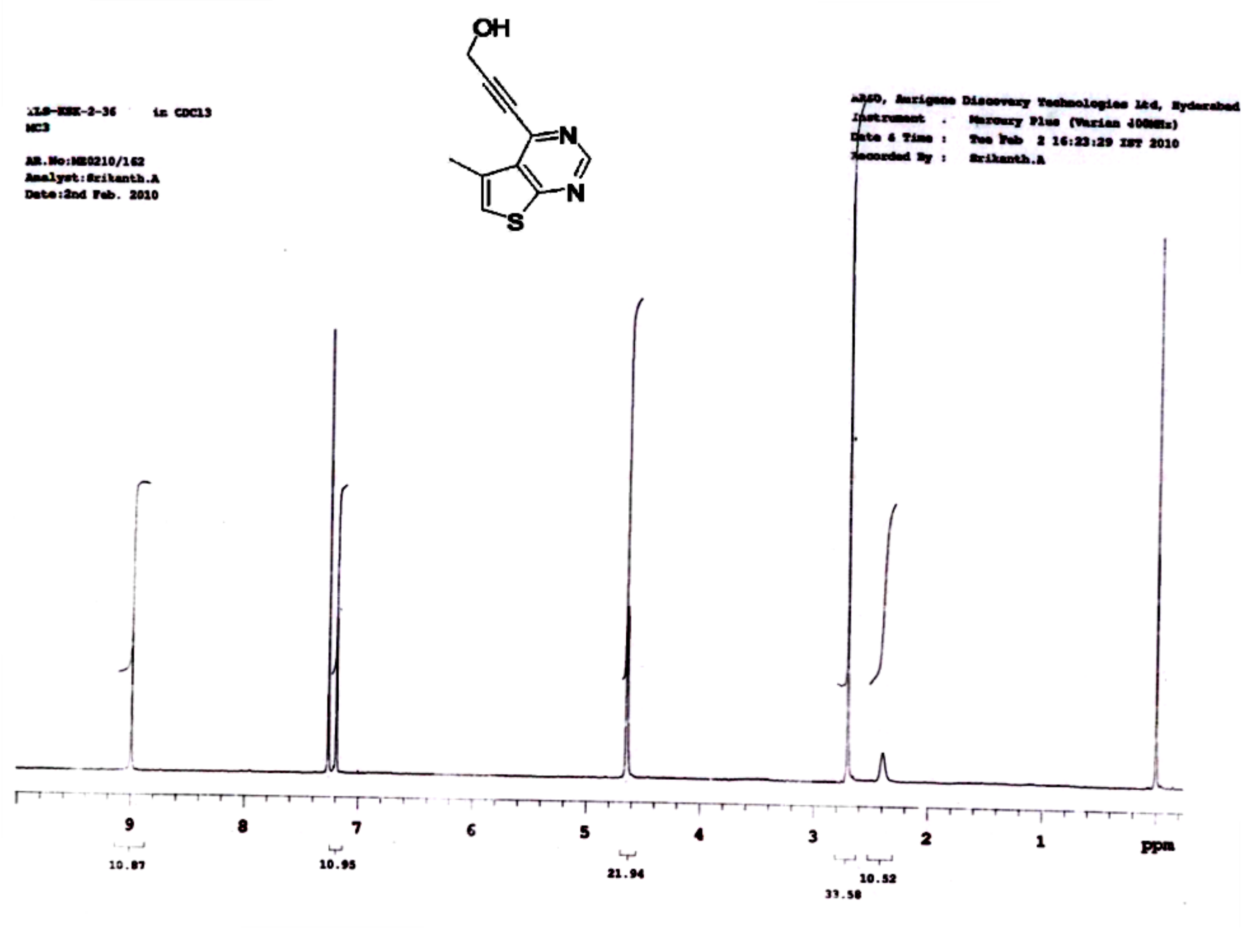

3h ^{13}C NMR (100 MHz, $CDCl_3$)

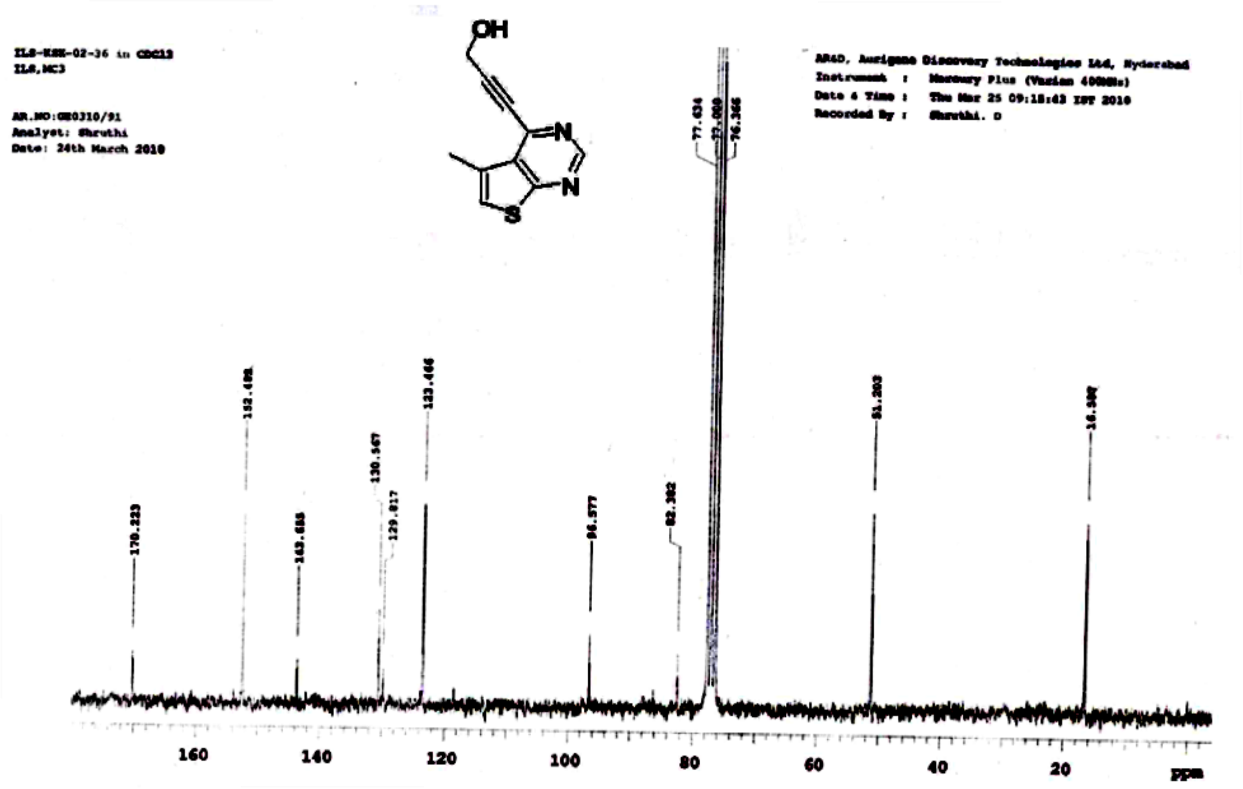

3i ^{1}H NMR (400 MHz, $CDCl_3$)

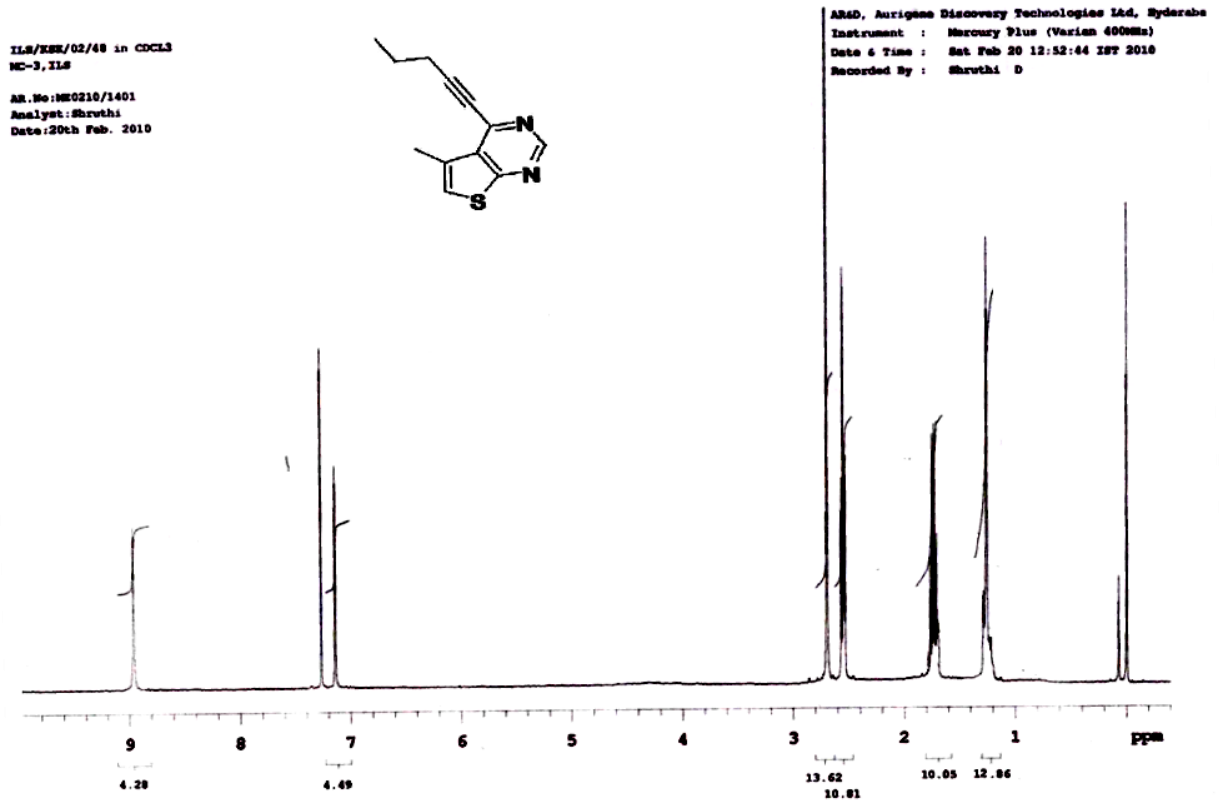

3i ^{13}C NMR (100 MHz, $CDCl_3$)

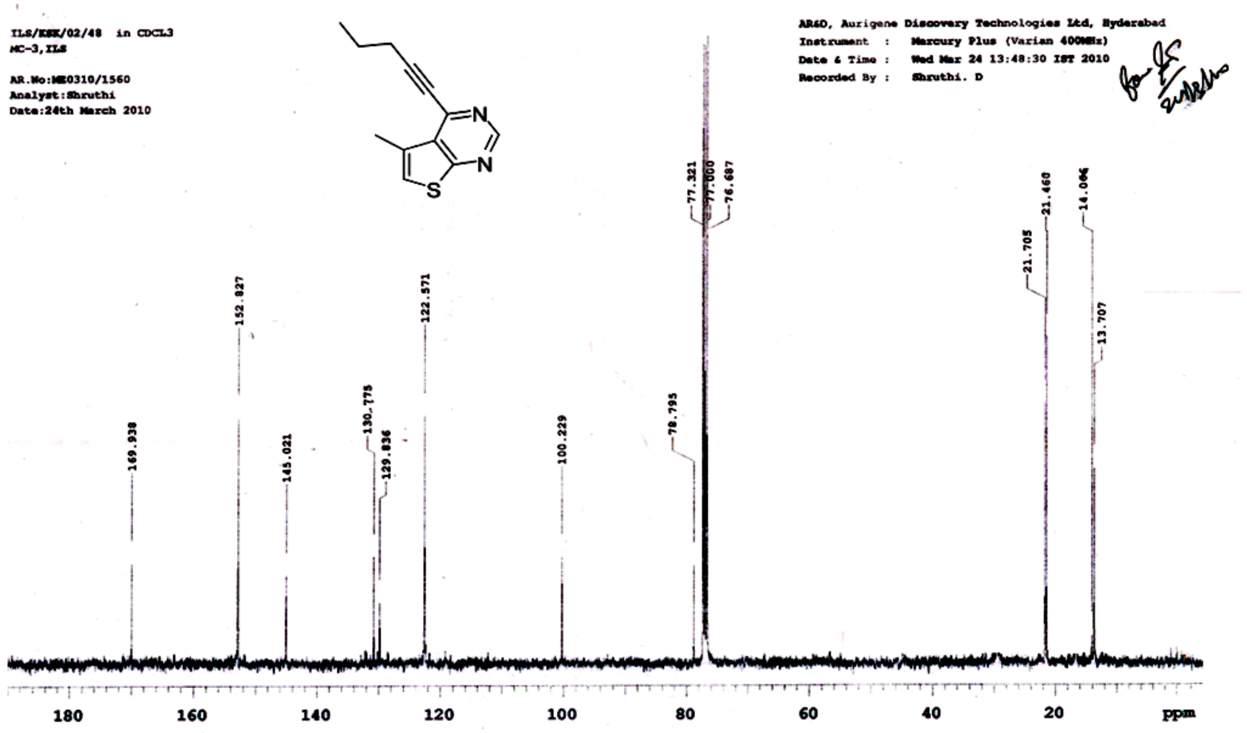

3j ^{1}H NMR (400 MHz, $CDCl_3$)

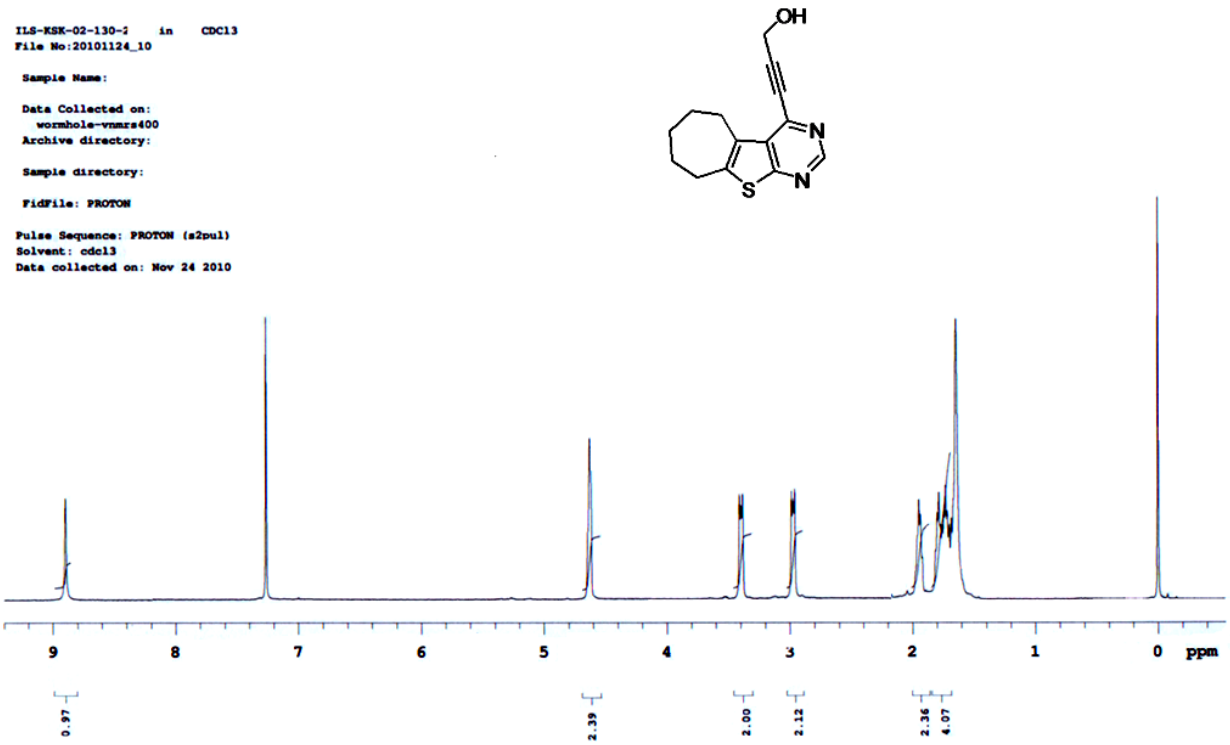

3j ^{13}C NMR (100 MHz, $CDCl_3$)

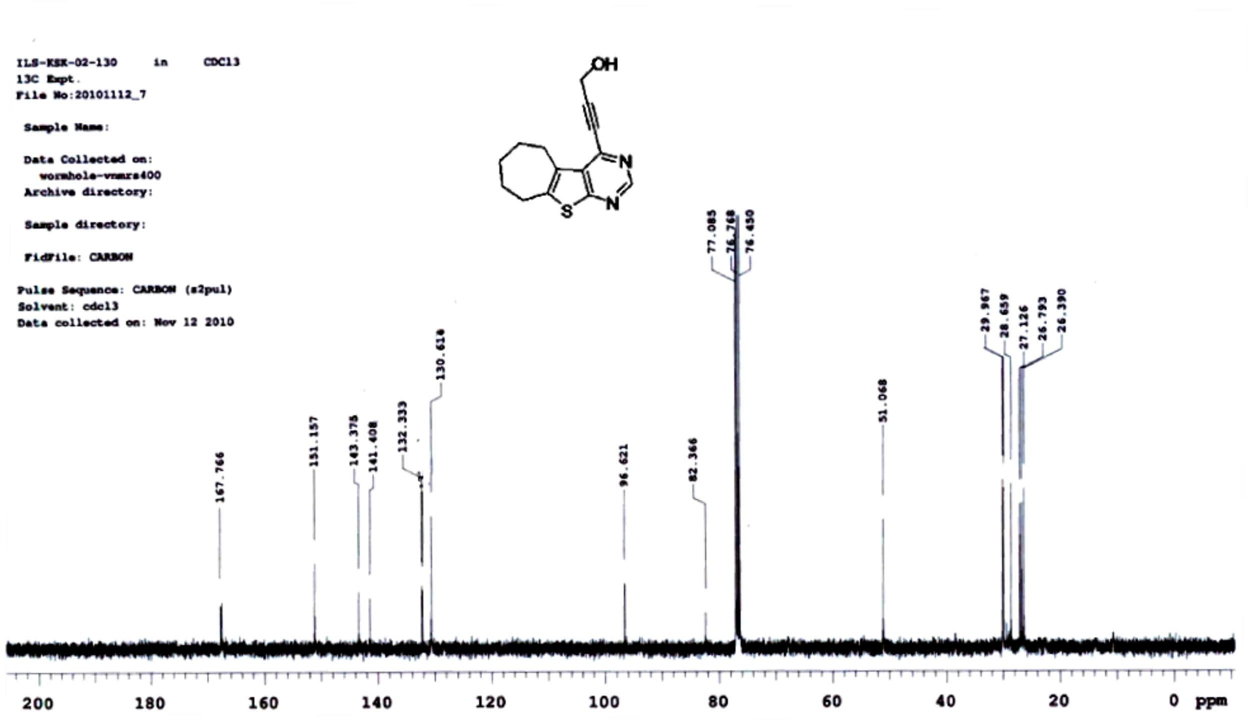

3k ^{1}H NMR (400 MHz, $CDCl_3$)

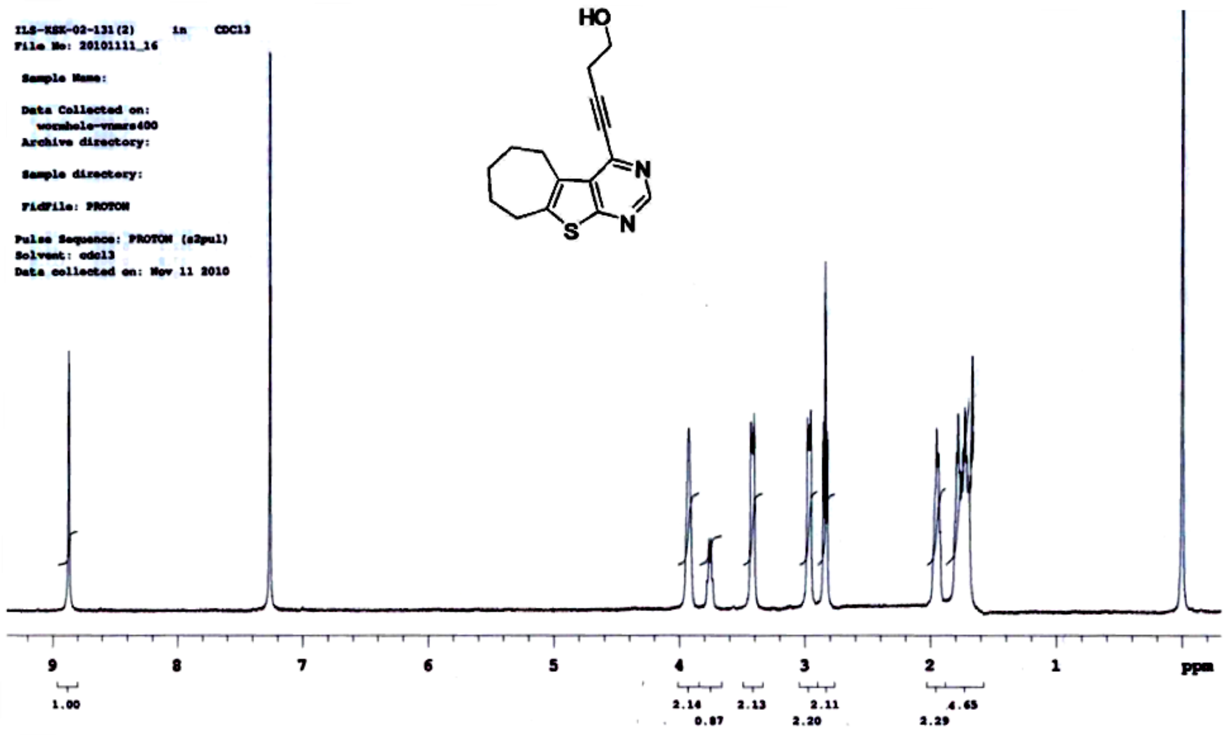

3k ^{13}C NMR (100 MHz, $CDCl_3$)

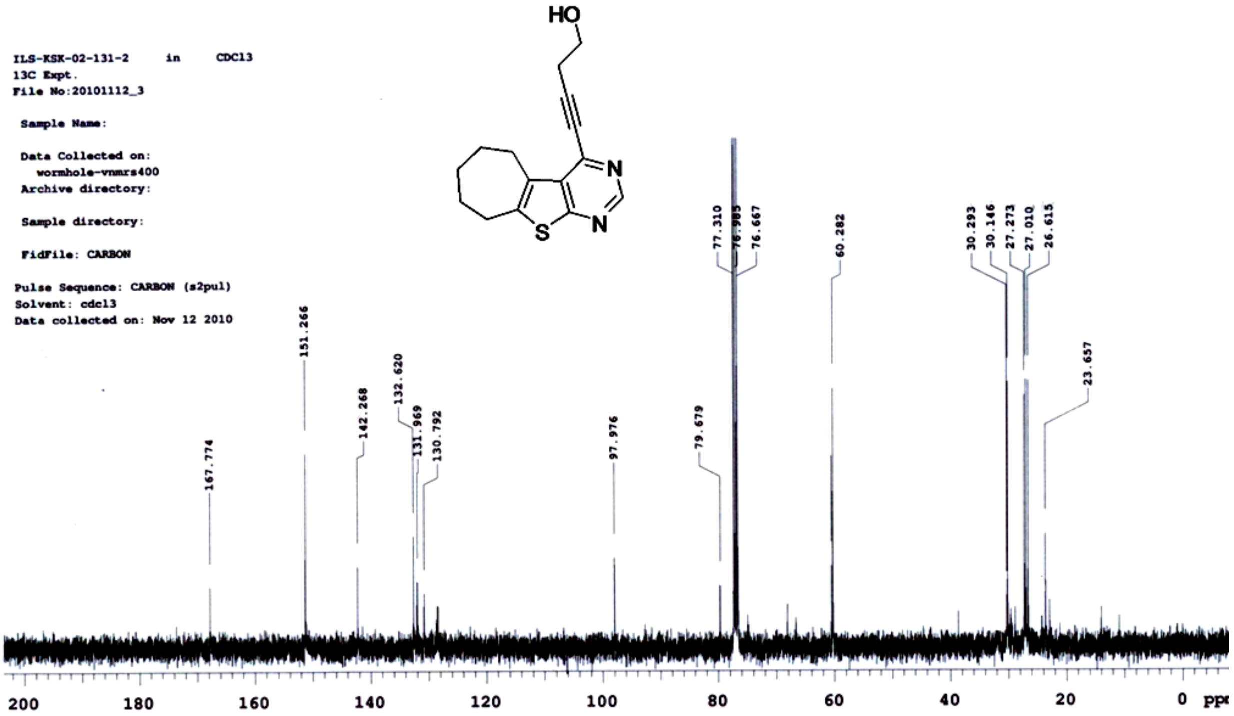

3l ^{1}H NMR (400 MHz, $CDCl_3$)

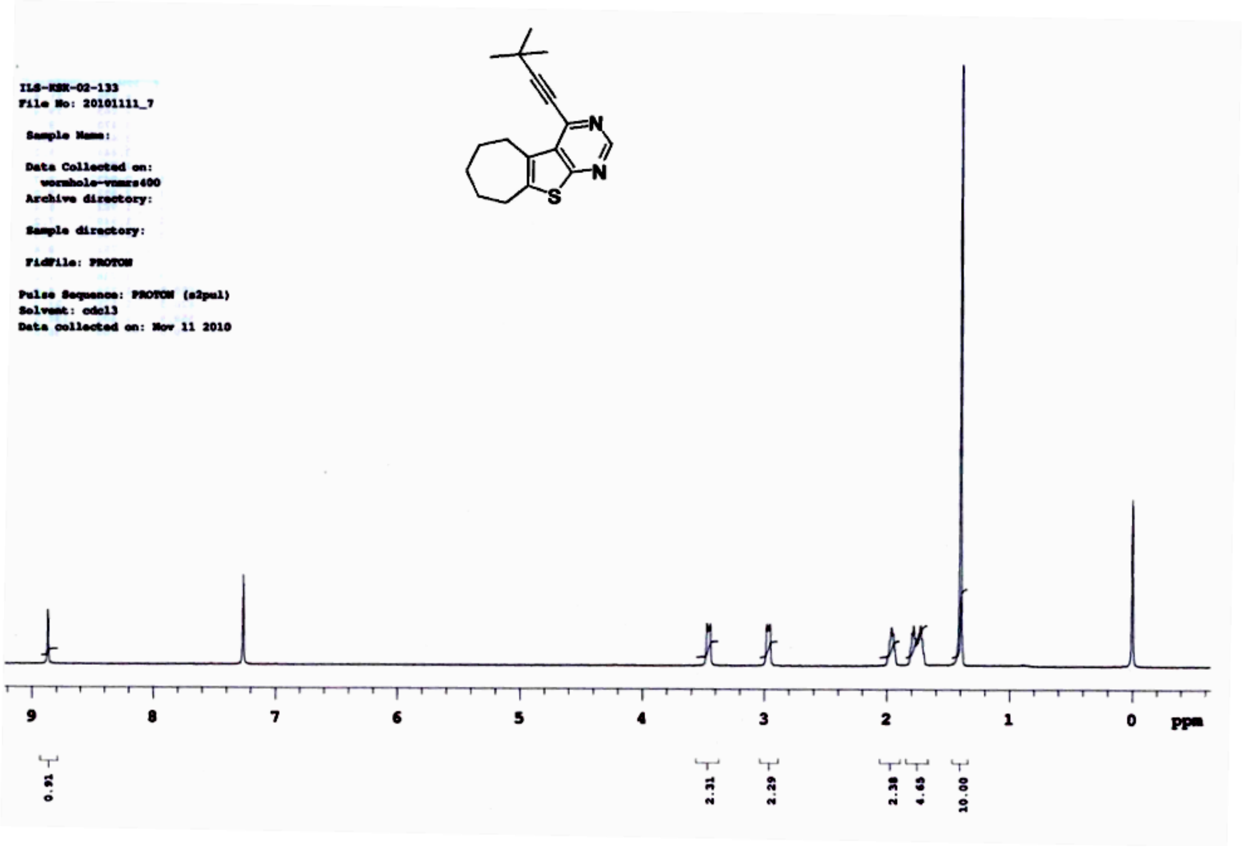

3l ^{13}C NMR (100 MHz, $CDCl_3$)

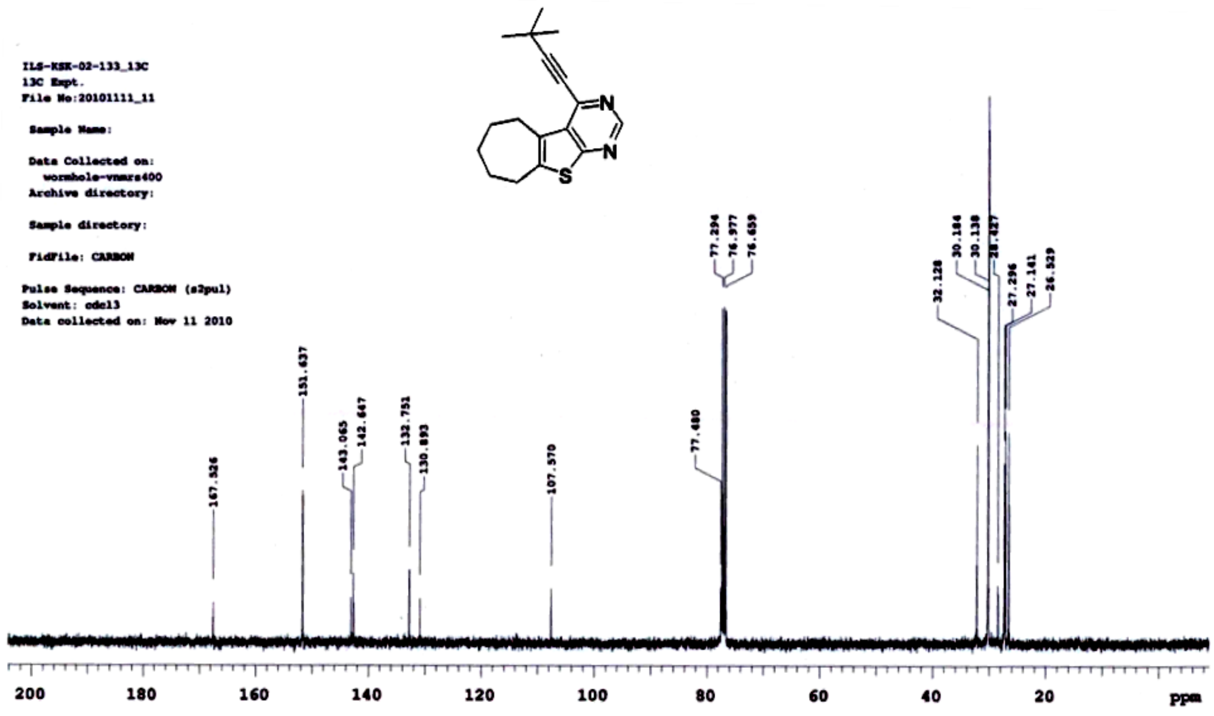

3m ^{1}H NMR (400 MHz, $CDCl_3$)

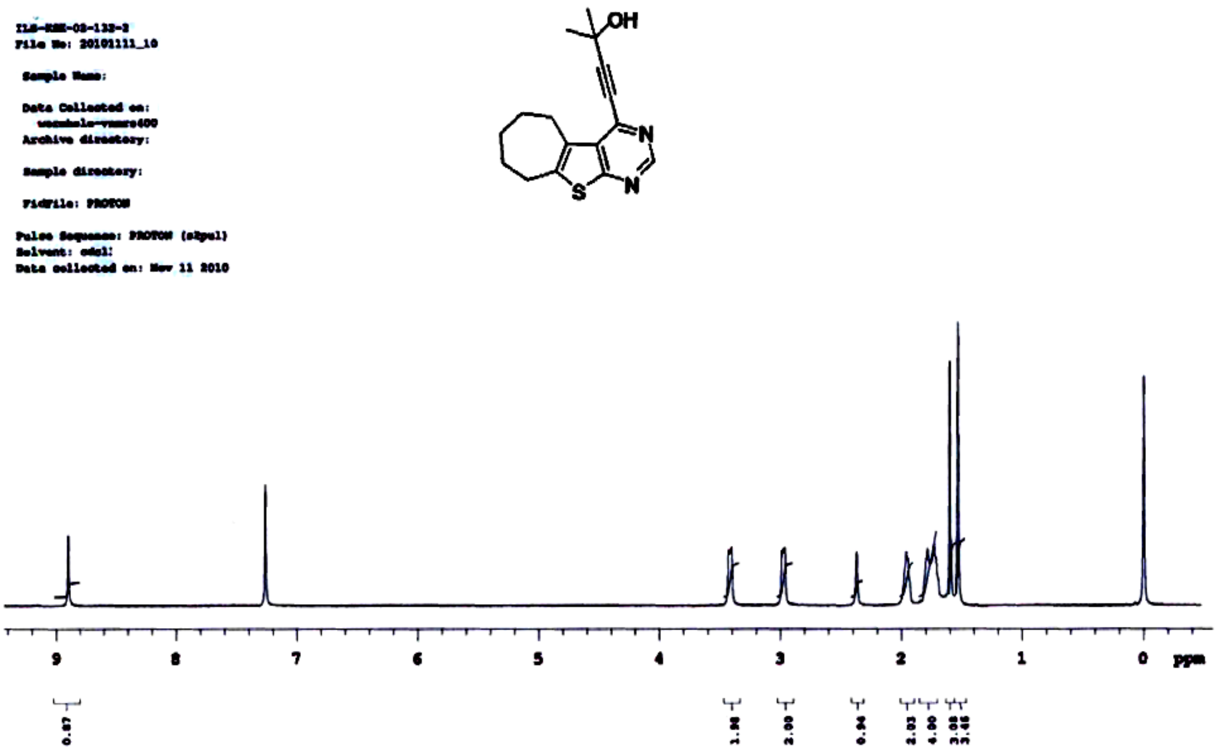

3m ^{13}C NMR (100 MHz, $CDCl_3$)

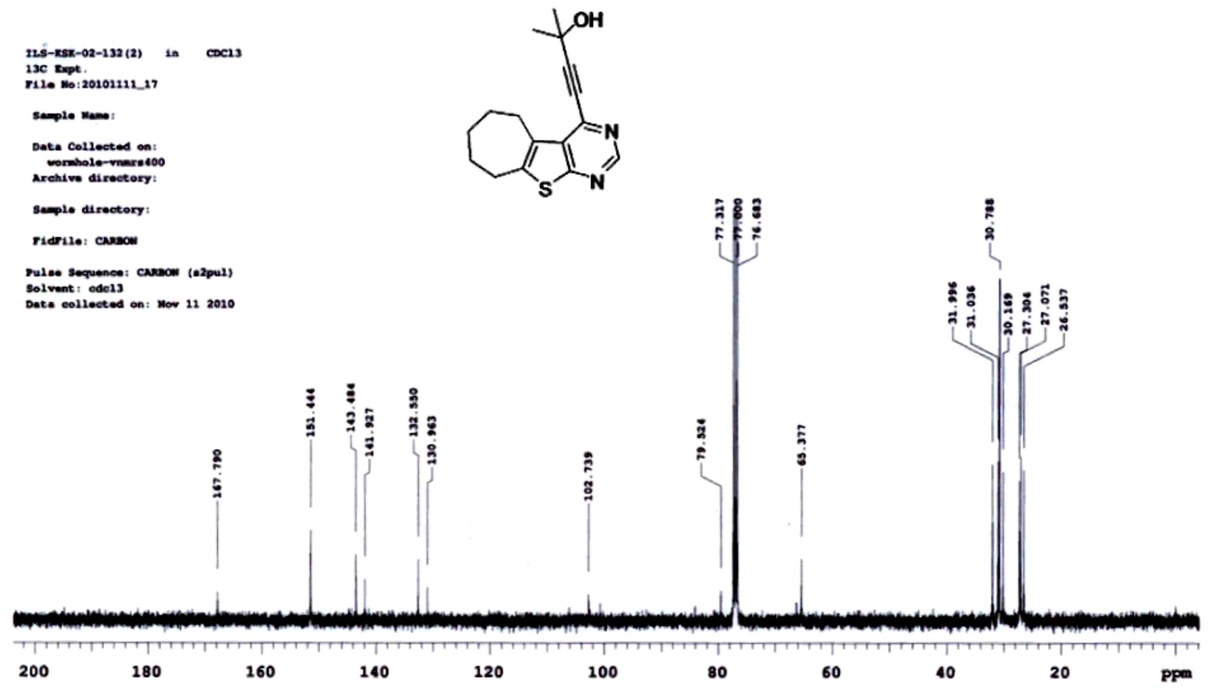

3n ^{1}H NMR (400 MHz, $CDCl_3$)

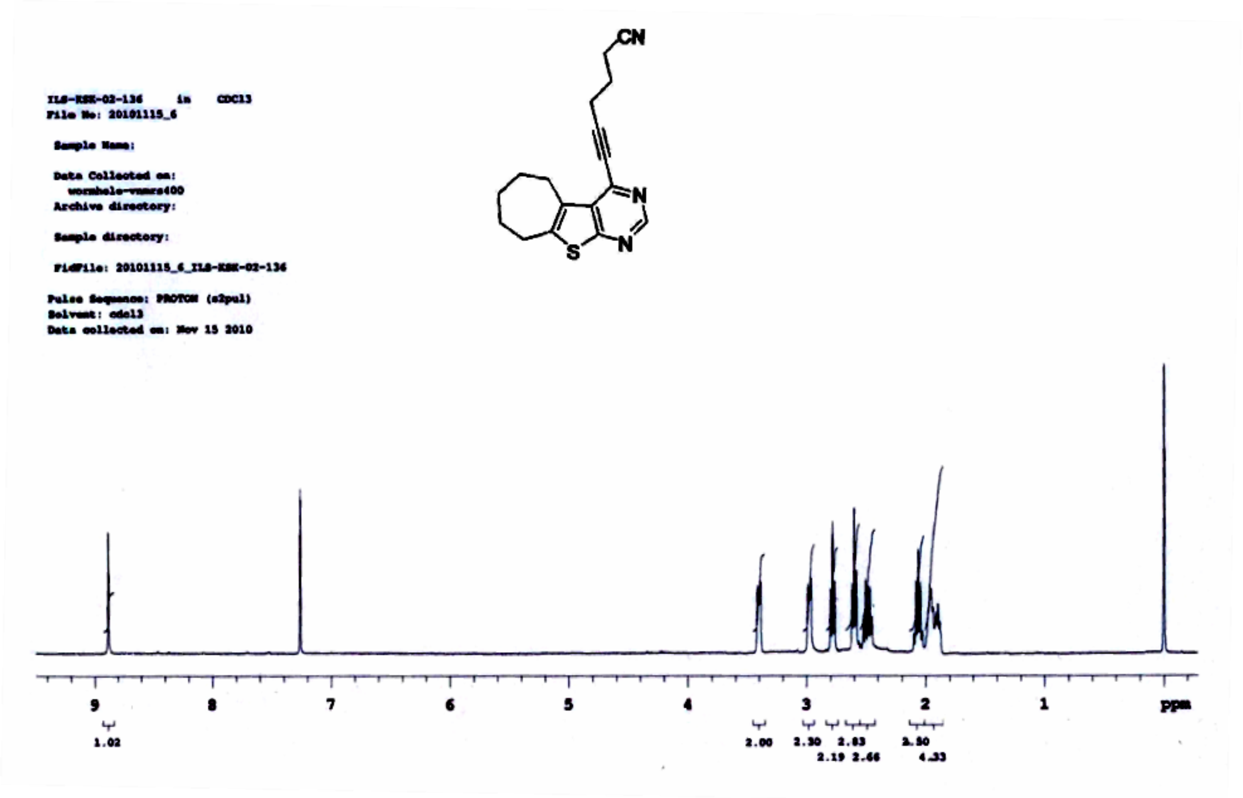

3n ^{13}C NMR (100 MHz, $CDCl_3$)

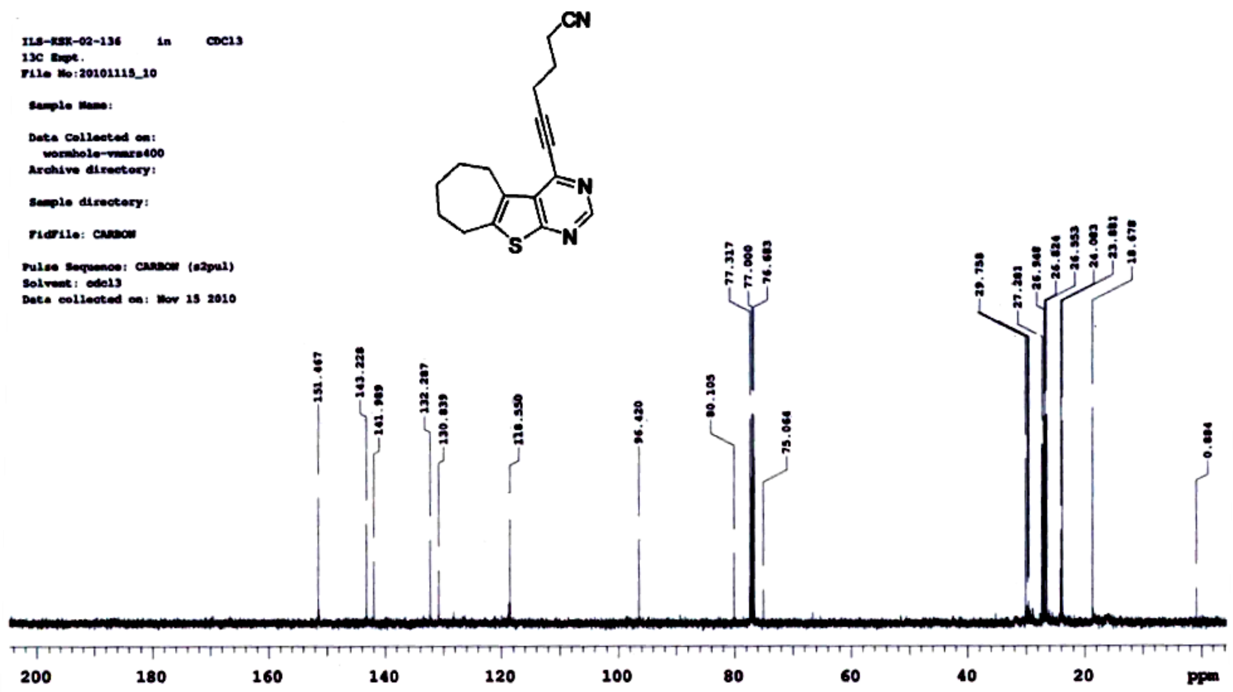

Supplement: File 2 — NMR spectra of compounds 3a–n. [file Beilstein_J_Org_Chem-07-338-s002.pdf]
